# A Fast Dissolution Pretreatment to Produce Strong Regenerated Cellulose Nanofibers via Mechanical Disintegration

**DOI:** 10.1021/acs.biomac.1c00466

**Published:** 2021-07-07

**Authors:** Juho Antti Sirviö, Matias Lakovaara

**Affiliations:** Fibre and Particle Engineering Research Unit, University of Oulu, P.O. Box 4300, 90014 Oulu, Finland

## Abstract

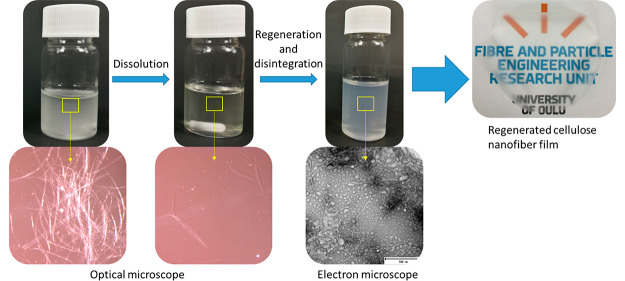

This study investigates a fast dissolution and regeneration pretreatment to produce regenerated cellulose nanofibers (RCNFs) via mechanical disintegration. Two cellulose pulps, namely, birch and dissolving pulps, with degree of polymerizations of 1800 and 3600, respectively, were rapidly dissolved in dimethyl sulfoxide (DMSO) by using tetraethylammonium hydroxide (TEAOH) as aqueous electrolyte at room temperature. When TEAOH (35 wt % in water) was added to the pulp–DMSO dispersion (pulp:DMSO and TEAOH:DMSO weight ratios of 1:90 and 1:9, respectively), 95% of the dissolving pulp and 85% of the birch pulp fibers dissolved almost immediately. Addition of water caused the regeneration of cellulose without any chemical modification and only a minor decrease of DP, whereas the crystallinity structure of cellulose transformed from cellulose I to cellulose II. The regenerated cellulose could then be mechanically disintegrated into nanosized fibers with only a few passes through a microfluidizer, and RCNF showed fibrous structure. The specific tensile strength of the film produced from both RCNFs exceeded 100 kN·m/kg, and overall mechanical properties of RCNF produced from birch pulp were in line with reference CNF produced by using extensive mechanical disintegration. Although the thermal stability of RCNFs was slightly lower compared to their corresponding original cellulose pulp, the onset temperature of degradation of RCNFs was over 270 °C.

## Introduction

Naturally derived materials and chemicals are currently recognized as sustainable alternative to the oil-based products used in everyday life.^1^ Despite many advantages of oil-based materials, such as plastics, their environmental friendliness is compromised due to the nonrenewability of their raw material, poor biodegradability, and toxicity. The sustainable use of naturally occurring components, such as cellulose,^2,3^ hemicelluloses,^4,5^ lignin,^6,7^ and starch,^8,9^ among others,^10−12^ in place of plastic could overcome the above-mentioned shortcomings.

Cellulose is the most abundant natural polymer and is widely available in nonedible plants.^13^ Therefore, cellulose is among the most relevant raw materials in plastic replacements as well as a starting point for a wide variety of novel products. Especially the nanosized cellulose, for example, cellulose nanofibers (CNFs) and crystals (CNCs), has been intensively studied to produce materials like strong self-standing films.^14^ The advantages of nanocellulose films compared with plastics are their very high mechanical strength and oxygen barrier properties, especially at moderate humidity.^15^

In nature, cellulose is produced as cellulose microfibrils or elemental fibrils, which are nanosized constituents of molecular cellulose.^16^ However, because of the strong hydrogen bonding ability, microfibrils are aggregated into large-sized macrofibers, and a significant high amount of energy is requested to liberate nanosized cellulose from these fibers. Nevertheless, we can lower energy consumption by pretreating natural fibers using mechanical, enzymatic,^17^ chemical,^18,19^ or solvent-based methods.^20,21^

The dissolution and regeneration is also known to be an efficient method in producing nonconventional nanosized cellulose materials.^22,23^ Upon the dissolution, the natural cellulose structure, that is, the microfibrillar order, is lost, which could lower the energy consumption during the mechanical disintegration, especially if material is not dried in between treatments. So far, solvents such as ionic liquids,^24^ deep eutectic solvents,^22^ and *N*-methylmorpholine *N*-oxide^25^ have been used to dissolve cellulose to produce nanomaterials. In addition, aqueous LiBr systems have been used to partially dissolve cellulose to liberate CNFs.^26^ Although systems like ionic liquids dissolve a substantial amount of cellulose (over 30 wt %),^27^ most of the above-mentioned solvents require long dissolution times and/or high temperature. In addition, apart from aqueous LiBr,^28^ there is scarcity of information about production of bulk materials, such as films, from regenerated cellulose nanomaterials.

In this study, we investigated the production of regenerated cellulose nanofibers (RCNFs) using novel solvent systems based on dimethyl sulfoxide (DMSO), which can dissolve cellulose rapidly in room temperature. DMSO is a common solvent used in cellulose dissolution and has been studied as a cosolvent with ionic liquids,^29^ where it helps decrease the viscosity of the solution.^30^ In addition, cellulose can be dissolved in DMSO in the presence of electrolytes, such as tetrabutylammonium acetate^31^ and fluoride.^32^ DMSO is produced from dimethyl sulfide,^33^ a byproduct of the Kraft process used for cellulose pulping, thus being a sustainable solvent. DMSO itself has relatively low toxicity; however, it should be noted that it penetrates skin, thus allowing solutes to pass into the living organism.^34^ Therefore, care should be taken when working with DMSO in the presence of toxic solutes.

In this study, DMSO was combined with aqueous tetraethylammonium hydroxide (TEAOH). TEAOH belongs to the class of aqueous tetraalkylammonium hydroxide ionic liquids that are studied for cellulose dissolution as such^35^ or together with additional components, such as urea.^36^ Tetraalkylammonium hydroxides are known to dissolve cellulose even at room temperature, and despite the apparent toxicity of alkyl cations, they are biodegradable. Moreover, it has been proposed that after use in dissolution and regeneration of biomass, they could be used as fertilizer.^37^ Previously, cellulose has been dissolved in DMSO by using aqueous tetrabutylammonium hydroxide (TBAOH). Here, dissolution of two cellulose pulps in DMSO using TEAOH at room temperature was investigated, and the production of RCNF was studied via mechanical disintegration of regenerated cellulose. The films were produced from RCNFs, and the mechanical and barrier properties of the films were compared to the films produced from traditional CNFs obtained by using mechanical disintegration.

## Materials and Methods

### Materials

Birch pulp (BP) and dissolving pulp (DiP) were obtained as dry sheets. The properties of pulps are presented in previous studies.^38,39^ DMSO and TEAOH (35 wt % in water) used for cellulose dissolution were obtained from VWR and Sigma-Aldrich, respectively. Deionized water was used for sample washing and dilutions.

### Dissolution of Cellulose

Cellulose pulp (1 g) was dispersed in DMSO (90 g) by mixing with magnetic stirring for 15 min at room temperature. Approximately 9.8 mL (1 g) of TEAOH was then added in one portion, and the mixture was stirred for 5 min. Cellulose was regenerated by adding 200 mL of water, after which the sample was filtered and washed with water until the suspension become neutral. The regenerated cellulose samples were stored at 4 °C.

To investigate the amount of dissolved cellulose, after the addition of TEAOH, the mixture was centrifuged at 10000 rpm for 10 min at 5 °C with a Beckman Coulter Avanti J-26 XPI (USA) centrifuge. Approximately 40 g of the upper layer was then removed from centrifuge tube, and cellulose was regenerated and washed as described above. Regenerated cellulose was then dried in an oven at 60 °C. The concentration of dissolved cellulose was calculated based on the dry mass of regenerated cellulose. For the reference, dissolution of cellulose pulps in a mixture of NaOH, urea, and water (7:12 :81 by weight) was investigated in a similar way described in the literature.^40^ The amount of dissolved cellulose was studied in a similar way described above.

Optical microscope images of cellulose pulp dispersion in DMSO before and after addition of TEAOH were acquired with a Leica MZFLIII microscope.

### Viscosity

The viscosity of the cellulose solutions in DMSO–TEAOH was investigated by using a TA Instruments Discovery HR-1 hybrid rheometer with cone–plate geometry (cone diameter of 40 mm and cone–plate angle of 1.999°). The measurements were conducted at 20 °C.

### Characterization of Regenerated Cellulose

Chemical characterization of cellulose pulps before and after dissolution was investigated by diffusion reflectance infrared Fourier transform spectroscopy (DRIFTS) using a Bruker Vertex 80v spectrometer (USA). The spectra were collected in the range 4000–600 cm^–1^ by using a resolution of 2 cm^–1^. The number of scans per sample was 40.

The average degree of polymerization (DP) was determined by using the limiting viscosity method according to the ISO 5351 standard.

X-ray diffraction (XRD) of the cellulose pulps before and after dissolution and regeneration and after fibrillation was conducted by a Rigaku SmartLab 9 kW rotating anode diffractometer (Japan) using Co Kα radiation (40 kV, 135 mA; λ = 1.79030 nm). The original and regenerated cellulose were first pressed into pellets with a thickness of 1 mm, and the nanofibrillated samples were measured from the self-standing films. Scans were taken over a 2θ (Bragg angle) range from 5 to 50° at a scanning speed of 10°/s by using a step of 0.5°. The crystallinity index (CrI) was estimated from Gaussian fitting of the XRD diffractograms using an OriginPro 2020 by dividing the sum of the crystalline peak areas with the sum of all peak areas.^41^

### Disintegration of Cellulose into Nanofibers

The nondried regenerated cellulose produced as described above was diluted to a concentration of 0.5 wt % and disintegrated by passing the sample three times at a pressure of 1000 bar through the 400 and 200 μm chambers of microfluidizer (Microfluidics M-110EH-30, USA). Because of the minor dilution of the samples during the disintegration, the dry matter content of fibrillated samples was determined gravimetrically. Samples prepared from regenerated BP and DiP were named RCNF1 and RCNF2, respectively.

Reference CNFs were produced from DiP by using combined disintegration with a friction grinder using a super mass colloider (Masuko MKCA6-2, Japan) and a microfluidizer (Microfluidics M-110EH-30, USA).^42^ At first, the sample (1.5% in water) was passed three times through −20 μm, twice through −40 μm, once through −60 μm, once through −80 μm, and twice through −90 μm with a rotating speed of the grinding stones of ∼1500 rpm. The sample was then further diluted to 0.5% and passed through a microfluidizer: once through 400 and 200 μm chambers at a pressure of 1000 bar and four times through 400 and 100 μm chambers at a pressure of 1500 bar.

### Preparation of Regenerated Cellulose Nanofiber Films

The RCNF films were produced via the filtration method. First, the RCNF dispersion with 0.33 g of dry matter was diluted into 100 g with water. Air bubbles were removed by sonication in an ultrasound bath (Elmasonic P, Elma Schmidbauer GmbH) at room temperature with 37 kHz and power of 100% for 10 min by using the degas mode. The dispersion was then filtered on a membrane (Durapore DVPP 0.65 μm, Merck Millipore Ltd., Ireland) in a negative pressure of ∼800 mbar. Filtration was stopped after the time difference between two consequent drops from the funnel was 30 s,^43^ and the samples were dried between two membranes by using a vacuum dryer (Karl Schröder KG, Germany) at 93 °C and at a negative pressure of 930 mbar for 10 min. Thus, the mass of the produced film was 60 g/m^2^. The reference CNF film was produced with the same method.

### Tensile Properties of Films

The tensile properties of the films were measured at 23 °C and a relative humidity (RH) of 50%; these conditions were used on the sample for at least 48 h prior to the measurement. The films were cut to sample strips with a diameter of 5 × 70 mm, and the average thickness was measured from the three random positions by using a thickness gage (Precision Thickness Gage FT3, Hanatek Instrument, UK). The tensile test was conducted by using a universal tensile machine (Zwick D0724587, Switzerland) with a 100 N load cell. During the test, the gage length was set to 40 mm, and the strain rate was set to 4 mm/min. A prestrain of 0.1 N was used.

### Scanning Electron Microscopy

The morphology of original and regenerated cellulose was characterized by using a FESEM (Zeiss Sigma Ultra plus, Germany) with an acceleration voltage of 5.0 kV. Samples were first dispersed in water at a concentration of 0.5% overnight and then filtrated on the membrane. Samples were sputter coated with platinum (high-resolution sputter coater, Agar Scientific, UK) before analysis using a sputtering time of 30 s and a current of 40 mA.

The morphology of RCNF films was observed with a SEM (Zeiss Zigma HD VP, Germany). The cross-section images were obtained from tensile test samples after rupture.

### UV/Vis Spectrometry

The transmittance of the RCNF and CNF films was measured in the wavelength range 200–800 nm by using a UV–vis spectrometer (Shimadzu, Japan). To ensure that the films were perpendicularly aligned against the incoming beam and to avoid wrinkling, the films were put between two quartz glass slides before they were set up in a cuvette stand.

### Thermogravimetric Analysis

The thermal property of the original cellulose pulp and cellulose nanomaterials was characterized by using a Netzsch STA 449F3 (Germany) thermogravimetric analyzer. Approximately 5 mg of dry sample was heated in aluminum oxide pan from 30 to 950 °C at a rate of 10 °C/min under air flow (dynamic air) at a constant rate of 60 mL/min. The first-derivative curves of the TGA (DTG) were calculated by using OriginPro 2019 software.

### Statistical Analysis

One-way analysis of variance (ANOVA) was conducted by using an OriginPro 2019 to determine the statistical significance (*p* < 0.05).

## Results and Discussion

### Dissolution and Regeneration of Cellulose Pulps

**Figure 1:**
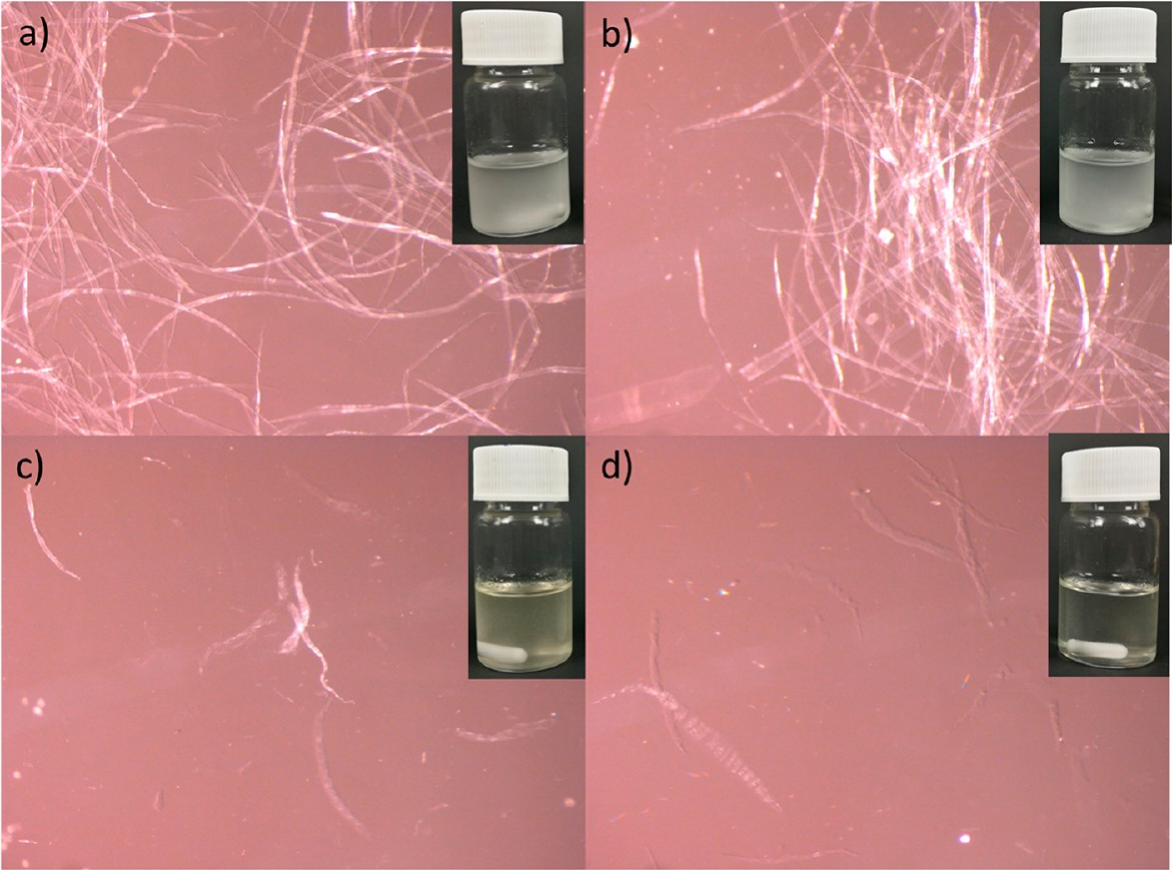
Microscopic images of cellulose pulp dispersed in DMSO: (a) BP and (c) DiP and directly after addition of TEAOH: (b) BP and (d) DiP (insets are photos of the solutions).

To produce RCNFs, the dissolution of two cellulose pulps was investigated: BP with a high hemicellulose content (24%) and DP (3600) and DiP with a low hemicellulose content (4%) and a medium DP (1800). Prior to the dissolution, cellulose pulp was dispersed in DMSO at room temperature to obtain a turbid suspension. After the addition of TEAOH, the mixture was significantly clearer and more viscose (Figure 1). However, although the solutions appeared as clear (especially in the case of DiP), few transparent, nondissolved fibers were observed in both cellulose pulps. It can be seen from the optical microscopic images that nondissolved fibers are swollen in DMSO–TEAOH. After the removal of nondissolved fibers via a centrifuge, it was observed that 95% and 85% of DiP and BP, respectively, were dissolved. Because of the high dissolution rate, further studies of regenerated cellulose were conducted without the removal of nondissolved cellulose fibers. For comparison, at the same concentration, the dissolution rates of DiP and DP in aqueous NaOH–urea solution were 20% and 19%, respectively.

**Figure 2:**
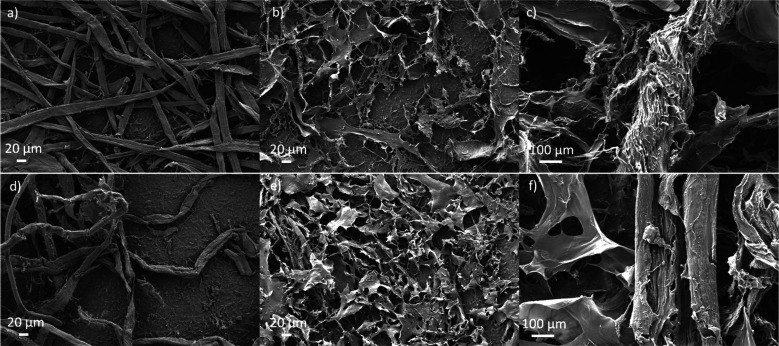
SEM images of original BP (a) and DiP (d) fibers and regenerated BP (b) and DiP (e) and higher magnification SEM images of nondissolved fibers in regenerated BP (c) and DiP (f).

Based on SEM images, most of the regenerated cellulose was uneven, flakelike material, indicating that the original fiber structure was disintegrated during the dissolution process (Figure 2). Few nondissolved fibers were observed with eroded and slightly disintegrated structure in both regenerated BP and DiP samples (Figure 2c,f). Therefore, although DMSO–TEAOH could not completely dissolve all the original cellulose fibers, solvent causes damage on the fiber’s structure.

The viscosity of the solution of DiP in DMSO–TEAOH was found to be slightly higher (0.5 Pa·s at a shear rate of 1 s^–1^) compared to the solution of BP (0.3 Pa·s at a shear rate of 1 s^–1^). Despite the lower DP of DiP compared to BP, the higher solubility might result in higher viscosity. Nevertheless, viscosities of solution were relatively low, and the solutions of DiP and BP in DMSO–TEAOH are poorly suitable for production of regenerated cellulose films or filaments. Because of the low viscosity, the solution is quickly mixed with antisolvent used to regenerate cellulose, and therefore production of an intact film or a filament is cumbersome. However, the low viscosity is advantageous for the purification step of regenerated cellulose, as low viscosity allows fast mass transport and thus limits the formation of gel-like material, where solutes (here DMSO and TEAOH) are trapped inside the regenerated cellulose.

**Figure 3:**
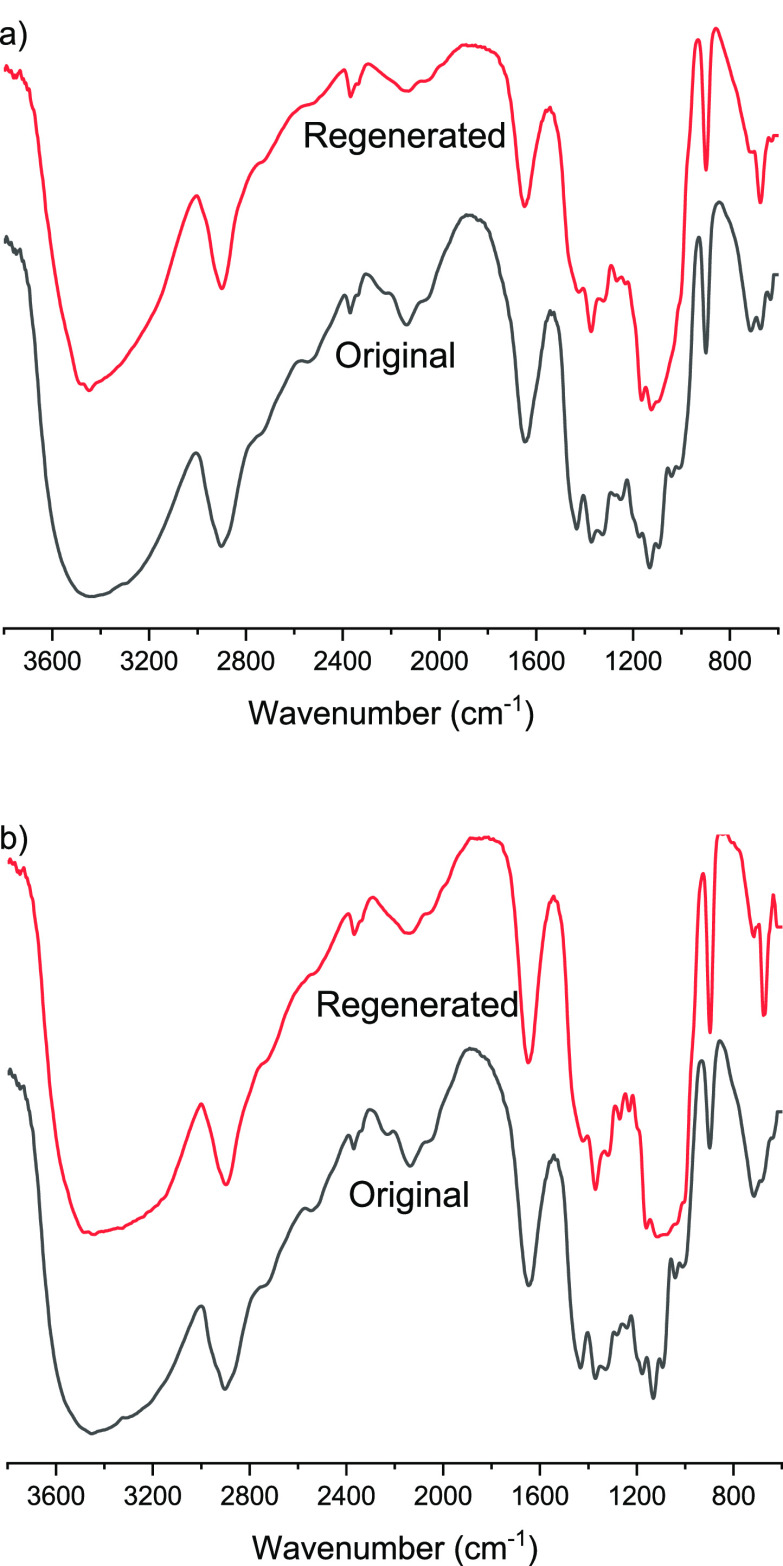
DRIFT spectra of BP (a) and DiP (b) before (original) and after regeneration.

During the dissolution, the crystallinity of original cellulose is lost, and the regeneration results in the rearrangement of ordered structure of cellulose to form cellulose II crystalline structure.^44^ After dissolution and regeneration, some small changes, especially at the fingerprint region, can be observed in DRIFT spectra, indicating that cellulose II was formed during the dissolution and regeneration of cellulose in the DMSO–TEAOH system (Figure 3). The intensity of the CH_2_ symmetric bending vibration in cellulose I at wavenumber of 1433 cm^–1^ decreased, and the local maximum appeared at a lower wavenumber of 1424 cm^–1^. The shift in CH_2_ symmetric bending vibration is due to the changes that occur in the vicinity of the hydroxymethyl group of cellulose as a result of the transformation of cellulose I crystallinity into cellulose II or amorphous cellulose.^45^ Furthermore, the shift of the C_1_ frequency from wavenumbers of 899 to 897 cm^–1^ as well as the appearance of a new shoulder in the OH region (3480 cm^–1^) indicated the changes in the crystallinity of cellulose during the dissolution and regeneration.^46^

**Figure 4:**
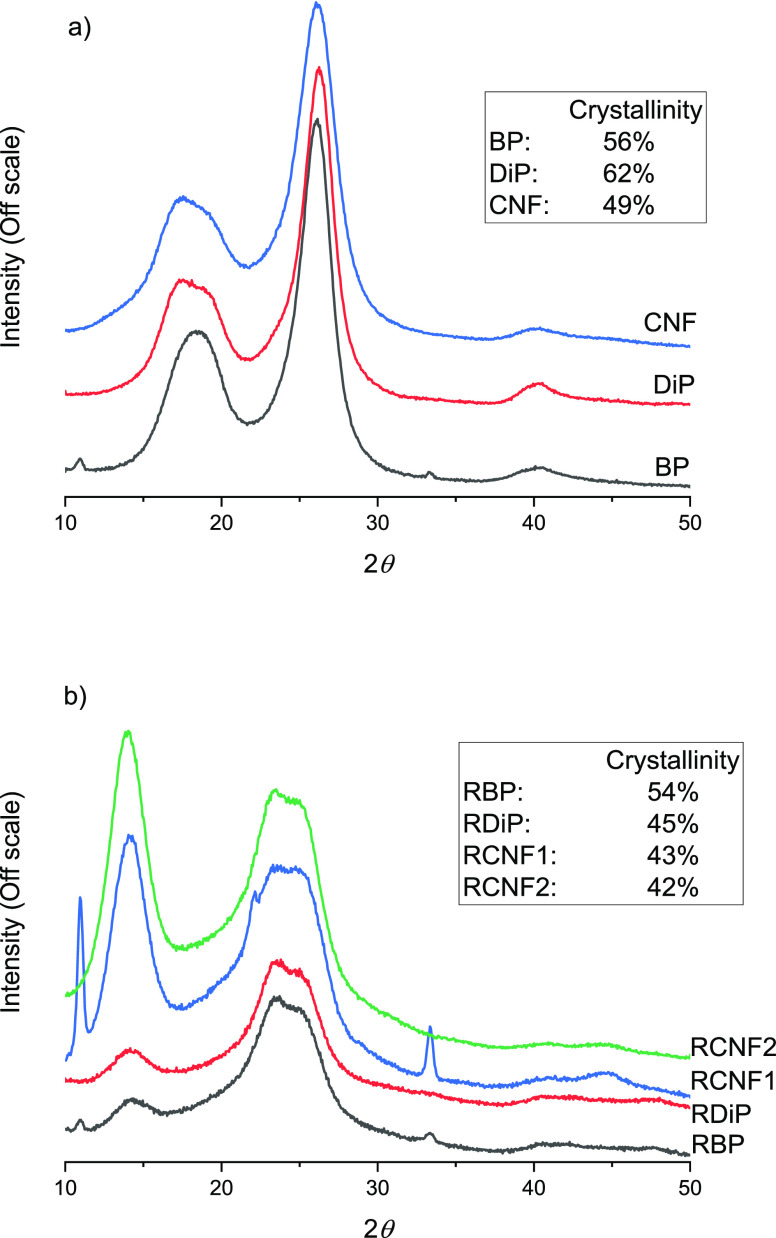
XRD diffractograms of (a) original pulps and CNF and (b) regenerated pulps and RCNFs (inset presents the crystallinity index of samples).

The changes in the crystalline structure of cellulose during the dissolution and regeneration from DMSO-TEAOH were further indicated in XRD measurements. Both BP and DiP exhibited typical cellulose I peaks around 17°, 19°, and 26° in XRD diffractograms (Figure 4).^47^ A weak signal was also observed around 40° (peaks around 10° and 34° are associated with sample holder^48^). Upon dissolution and regeneration, the peak of cellulose I disappeared, and new peaks were observed around 14°, 24°, and 25°. The new peaks can be attributed to the cellulose II crystalline structure.^47^

The slight decrease in the DP of both cellulose pulps was observed after dissolution and regeneration using DMSO–TEAOH, and DPs of regenerated BP and DiP were 3100 and 1500, respectively. The decrease in DP contradicts the previous studies using DMSO with TBAOH, where a minor increase in DP was reported.^49^ The increase in DP was associated with radical formation from DMSO in the presence of a strong base. In the case of TBAOH, the maximum radical activity was observed at a concentration of 20%, almost 2 times higher compared to the concentration of TEAOH in this study (11%). Therefore, it can be assumed that the presence of a radical in DMSO–TEAOH is small, and no radical-induced recombination of cellulose chains took place. The small decrease in DP in the case of DMSO–TEAOH might be due to the strong alkaline condition.^50^

The dissolution of cellulose in DMSO in the presence of tetraalkylammonium hydroxide is previously assumed to be the result of the combination of the electron donor–acceptor complexation and radical reaction.^49^ Although more studies regarding the dissolution mechanism and dissolution limits (e.g., maximum amount of cellulose in solution) of cellulose in DMSO–TEAOH systems should be conducted, it is likely that dissolution is mostly due to the hydrogen bonding or deprotonation of cellulose with hydroxide ion resulting in the dissolution of cellulose in DMSO due to the electrostatic repulsion between cellulose chains. The cation in tetraalkylammonium hydroxide systems is proposed to interact with carbon in position 1 in a cellulose ring by either electrostatic or van der Waals forces.^51^ As stated previously, the radical formation in DMSO–TEAOH was assumed to be minor and therefore does not take part in the dissolution process to a large extent.

### Nanofibrillation of Regenerated Cellulose

**Figure 5:**
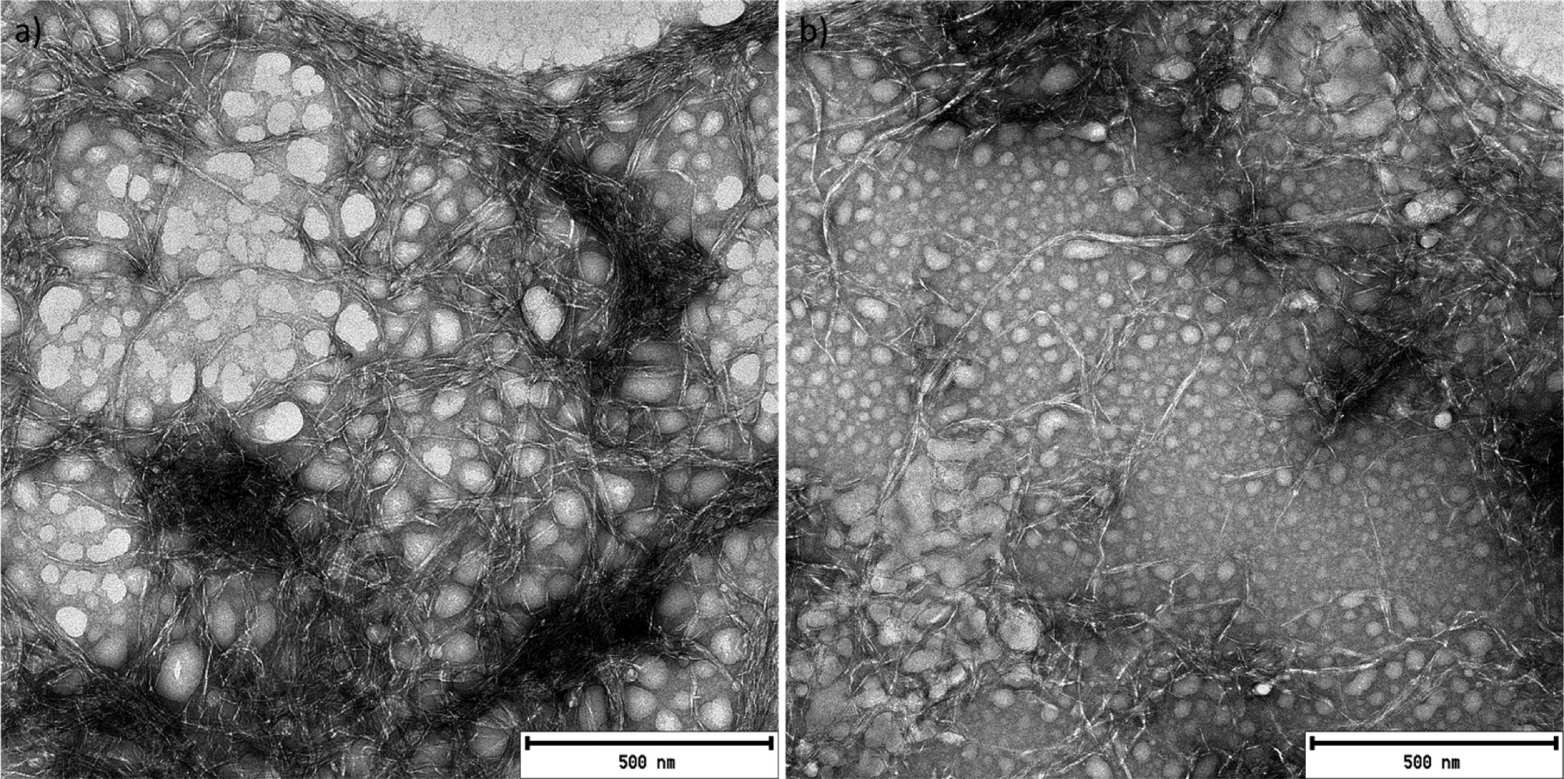
TEM images of RCNF1 (a) and RCNF2 (b) (round circles are due to the sample preparation, most likely due to the air bubbles trapped in the cellulose solution).^54^

Both of the regenerated cellulose samples passed easily through a microfluidizer to form slightly gel-like and turbid suspensions (Figure S1), similar to the CNFs produced from nonchemically modified cellulose. Based on the TEM imaging, RCNFs formed a weblike structure, analogous to the more traditional CNFs (Figure 5a,b) (more TEM images are presented in Figures S2 and S3). The weblike structure results from the physical cross-linking and entanglement and is a result of the long length and the lack of repulsion forces (e.g., electrostatic) between fibers. Because of the weblike structure, the length of the RCNFs could not be measured. On the other hand, the widths of nanofibers of RCNF1 and RCNF2 were 6.9 ± 4.9 and 5.9 ± 2.8 nm, respectively. The width values of the RCNFs were similar to those previously obtained from mechanical disintegration of chemically modified cellulose fibers,^52^ although CNFs with a diameter around half of the size of RCNFs can be produced by using methods such as (2,2,6,6-tetramethylpiperidin-1-yl)oxyl-mediated oxidation.^18^ Previously, CNFs produced from mercerized cellulose fibers (i.e., fiber with cellulose II crystalline structure) were obtained with a width of 15–100 nm after mechanical disintegration.^53^ The reference CNFs produced from DiP by mechanical disintegration and microfluidization had an average nanofiber width 10.4 ± 5.4 nm (TEM images of reference CNFs are presented in Figure S4).

Although no statistical difference was observed between the widths of the two RCNF samples, it appears that regenerated cellulose from DiP formed nanofibers with more narrow size distribution (a histogram of the widths is presented in Figure S5). Most of the RCNF2 fibers had a width around 5 nm. Minor amounts of large fiber bundles or aggregates and smaller few nanometer fibers were observed. On the other hand, RCNF1 contained a large amount of small (∼2 nm) fibers. In addition, there appeared to be more large (∼10–20 nm wide) fiber bundles in RCNF1 compared with RCNF2. It could be that the presence of hemicellulose, nondissolved fibers, and cellulose DP has an impact on the fibrillation efficiency of the regenerated cellulose. The hemicelluloses are known the help in the nanofibrillation of natural cellulose fiber by creating steric and electrostatic repulsion between fibers, thus allowing the production of few nanometer nanofibers.^55^ On the other hand, nondissolved cellulose fibers can be more resistant to the mechanical disintegration compared to dissolved and regenerated cellulose particles, resulting in a higher amount of large aggregates in the case of RCNF1. Furthermore, cellulose with a longer molecular chain can form more tightly packed structure upon regeneration, thus requesting more energy to disintegrate into even-sized nanofibers.

Previously, the use of an acidic deep eutectic solvent resulted in the formation of short (few tens of nanometers) fiberlike nanoparticles after mechanical disintegration of regenerated cellulose.^22^ The formation of short fibers was associated with hydrolysis of cellulose during dissolution, resulting in the formation of short molecular chain cellulose. As was mentioned previously, the use of DMSO–TEAOH only resulted in a minor decrease in the DP of cellulose. Thus, the cellulose molecules have a long chain length, and they are highly aggregated by hydrogen bonding. Therefore, the mechanical disintegration resulted in the formation of long nanofibers. Previously, precipitation of cellulose from ionic liquid resulted in the formation of fiberlike aggregated nanomaterials, whose dimensions could be decreased by homogenization.^56^ Most of the other studies have reported the formation of spherical-shaped regenerated cellulose nanoparticles^23,57−59^ or nanostructured gels.^25^

### Regenerated Cellulose Nanofiber Films

**Figure 6:**
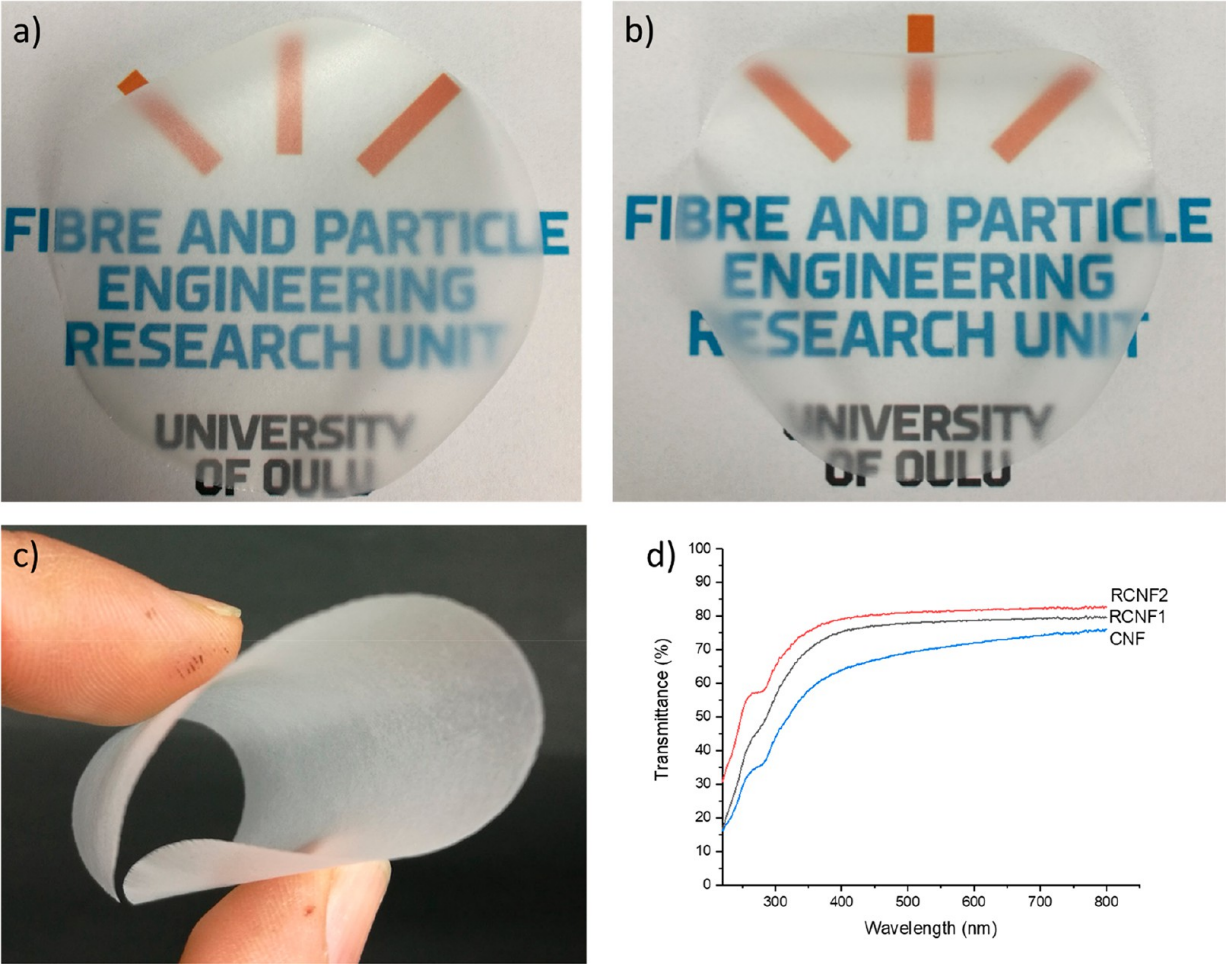
Photograph of films from RCNF1 (a) and RCNF2 (b) demonstrating their transparency. Bent CNF1 film (c). UV/vis transparency of the RCNF and CNF films.

One of the main advantages of cellulose-based nanofibers is that they can form strong filmlike structure by using the water-based filtration method, similar to that used in papermaking, and could thus enable the continuous large-scale production of nanomaterial films. Previously, the regenerated cellulose nanomaterials are obtained as spherical^23^ or very short fiberlike particles,^22^ and it can be assumed that they do not form an entangled network, thus resulting in the formation of brittle film structure, similar to the CNCs.^60,61^ On the contrary, RCNFs appeared as long fiberlike materials forming weblike structure, and by use of the filtration method, both RCNFs formed self-standing films that were slightly opaque and easy to handle (Figure 6) (a photograph of the reference CNFs is presented in Figure S6).

The light transparency of the self-standing films is essential on many applications as it allows the visual quality control of consumables as well as high efficiency of solar cells. The visible light transparency of the both RCNF films was over 70%, and there was around 3% unit difference on benefit of RCNF2 in the whole visible light range (Figure 5d). Compared to reference CNFs films, both RCNF films exhibited higher transparency at the whole measurement range. At a wavelength of 800 nm, the transmittance of RCNF1 and RCNF2 was around 4% and 5% units higher, respectively, compared to CNFs. At a wavelength of 400 nm, the differences were 11% and 15% units. Therefore, the UV/vis measurement further demonstrated that regenerated cellulose can be disintegrated into nanofibers with relatively uniform size (i.e., minor amount of large particle causing blocking of light), and the transparency properties of the RCNF films are in line or even slightly higher compared to traditional CNFs. It should be noted that by using chemical modification, such as oxidations, films with higher transparency can be produced.^62^

**Figure 7:**
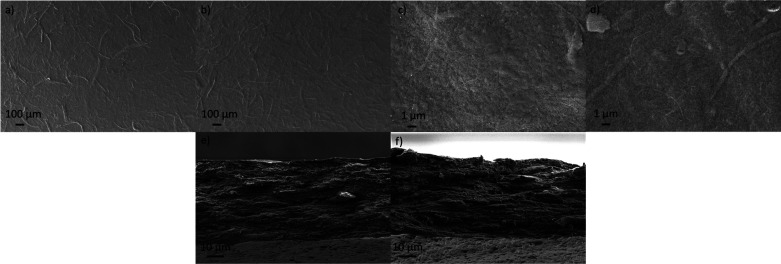
SEM images of surface and cross sections of RCNF1 (a, c, and e) and RCNF2 (b, d, and f) films.

The surface SEM images of RCNFs films showed that both of them contained some larger cellulose fibers embedded in the nanofiber network (Figure 7). Upon comparison of these two films, it appears that RCNF2 contained a lesser amount of larger, nondisintegrated fibers. This might be due to the higher dissolution rate of DiP in DMSO–TEAOH compared to BP. The larger fibers in BP are most likely from original cellulose pulp fibers that are not or are only partially nanofibrillated due to the relative mild mechanical treatment. The surface of both films showed some decree of porosity (Figure 7c,d), most likely due to the heterogeneity in fiber size, resulting in the formation of cavities that are not entirely filled by the smaller nanofibers. In addition, both films exhibited layered cross section, although layers are not so prominent in CNF2 films.

Good mechanical properties, such as tensile strength, are fundamental for cellulose nanomaterials films since they are expected to be exposed to various mechanical stress during their use (e.g., as packaging materials). As stated above, both RCNF films were easy to handle, and they could be bent, similar to more common types of CNFs (Figure 6c). The specific tensile strength of both RCNF films was over 100 kN·m/kg (Table 1), being well above common oil-based plastics like polypropylene (28–44 kN·m/kg)^63^ and poly(lactic acid) (43 kN·m/kg),^64^ a well-known bioplastic. The mechanical properties of BP-derived RCNF1 film were higher compared to the RCNF2 film, likely due to the higher DP of BP compared to DiP. The notable exception was in the specific yield strength, as there was no static difference between two samples. A similar specific yield strength indicates that both RCNF films tolerate similar force before enduring permeated deformation; that is, the original form of the samples is not regained when force is removed. After the yield point, the RCNF1 showed longer elongation and thus was subjected to more extended strain hardening, typical to fiberlike cellulose nanomaterials.^65^ Therefore, the specific tensile strength of RCNF1 was higher than that of RCNF2.

**Table 1:** Density and Mechanical Properties of RCNFs and Reference CNF Films^a^

sample	density (g/m^3^)	specific tensile strength (kN·m/kg)	specific tensile modulus (GN·m/kg)	work capacity (kJ/kg)	strain at break (%)	specific yield strength (kN·m/kg)	yield strain (%)	tensile strength (MPa)	tensile modulus (GPa)
RCNF1	1.02	131 ± 5^a^	8.3 ± 0.3^a^	5.3 ± 0.3^a^	5.5 ± 0.2^a^	93.7 ± 2.7^a^	1.3 ± 0.0^a^	133 ± 5^a^	8.4 ± 0.3^a^
RCNF2	1.04	104 ± 3^b^	7.4 ± 0.2^b^	3.1 ± 0.2^b^	4.0 ± 0.2^b^	86.3 ± 2.3^a^	1.3 ± 0.0^a^	109 ± 3^b^	7.7 ± 0.2^a^
CNF	1.25	131 ± 6^a^	8.6 ± 0.2^a^	4.8 ± 0.7^a^	5.0 ± 0.5^ab^	91.2 ± 2.8^a^	1.1 ± 0.0^b^	164 ± 8^c^	10.8 ± 0.2^b^

a Different superscript letters within the same column are significantly different at the 0.05 level based on the one-way ANOVA.

In the literature, a wide range of mechanical properties of CNF films have been reported with specific tensile strength ranging from around 100 to over 200 kN·m/kg.^14,66,67^ The specific tensile strength, modulus, yield strength, and strain of film of CNF produced for DiP by intensive mechanical disintegration (combined disk grinding and microfluidization; see the Materials and Methods section) were similar to that of RCNF1 and only slightly above the values obtained for RCNF2. The comparable mechanical properties of RCNFs with CNFs show that dissolution and regeneration are suitable pretreatment to produce strong bulk material, such as films. Nevertheless, as was seen in SEM images, the RCNF films exhibited slightly porous structure, and their densities (around 1 g/m^3^) were significantly lower compared to the CNF film (1.25 g/m^3^). Therefore, the tensile strength and modulus of CNF film were higher compared to RCNF films and similar to those reported in the literature.

Different regenerated cellulose-based films with high mechanical strength have been reported. Commercially available cellophane has been reported to have tensile strengths in the range 50–120 MPa, being similar to those of RCNF films.^68^ Furthermore, cellulose films regenerated from *N*,*N*-dimethylacetamide–LiCl and aqueous LiOH–urea systems with tensile strengths of 170 MPa^69^ and 263 MPa,^70^ respectively, have been reported, being notably higher compared to RCNF films. Therefore, there is still room for improvement of the mechanical properties of RCNF films. Nevertheless, fast room temperature dissolution, relatively easy washing steps, and simple film preparation using vacuum filtration of RCNFs are advantageous compared to the many regeneration processes reported in the literature.

Although the mechanical properties of RCNF2 film were slightly lower compared to the CNF film, having same raw materials (i.e., DiP), the production of CNF requests two types of mechanical treatment, that is, grinding and microfluidization. The grinding step was necessary during the production of CNF, as DiP could not be passed through the microfluidizator. On the other hand, regenerated DiP was easily processed with microfluidization after the fast room temperature dissolution and regeneration process. Therefore, DMSO–TEAOH pretreatment is a potential pretreatment method for cellulosic nanomaterial production. However, it should noted that more research is needed to optimize the dissolution process as well as to calculate the energy consumption of whole process (e.g., production of chemicals, washing steps, and chemical recycling) for proper comparison between different cellulosic nanomaterials. Especially the recycling of the solvent system is crucial as the large consumption of solvent chemicals is both economically and ecologically nonsustainable. TEAOH together with urea is demonstrated to the recyclable solvent for cellulose,^36^ and DMSO–ionic liquid systems have been used as a recyclable solvent for cellulose.^71^ The simple evaporation of water from the washing system could facilitate the recycling of the DMSO–TEAOH system. However, as cellulose was first dispersed in pure DMSO, removal of TEAOH from washing liquor by using ion exchange could be a suitable method for separation of two solvent components, which could then be used for further dissolution. The recycling as well as the dissolution mechanism will be investigated in future studies.

### Thermal Properties of Regenerated Cellulose Nanofibers

**Figure 8:**
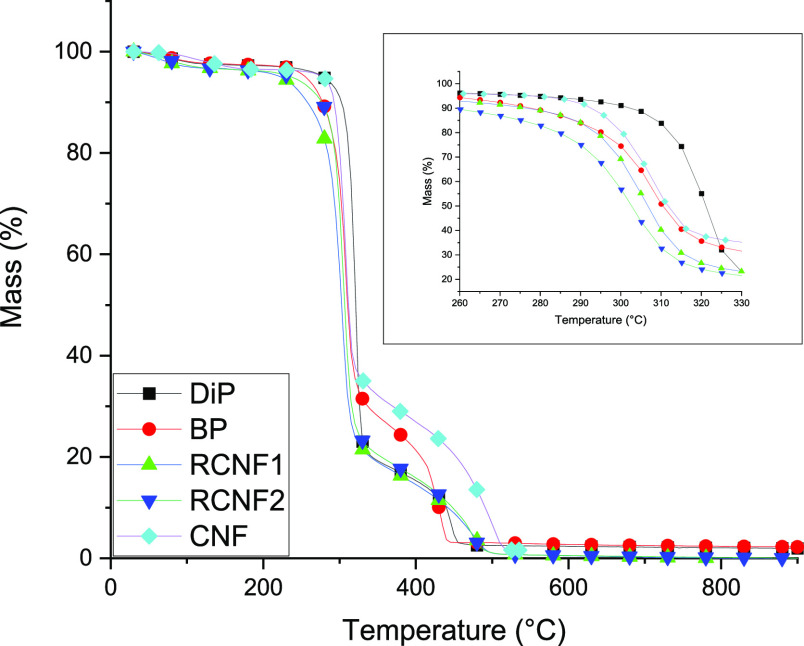
TGA curves of starting cellulose pulps, RCNFs, and CNF as reference (inset represents the onset temperature region of the samples).

Pure cellulose has relatively good thermal properties, making it suitable material for application requesting elevated temperature. However, chemical and physical alteration of the cellulose structure can have a notable effect on the thermal behavior of cellulose. Therefore, the thermal properties of original cellulose and RCNFs were investigated by TGA (Figure 8). In the presence of oxygen (at dynamic air atmosphere), the onset temperature of degradation of BP and DiP was at 280 and 301 °C, respectively (low mass loss in all the samples around 100 °C was due to the evaporation of water). The lower onset temperature of BP is due to the presence of substantial higher amount of hemicelluloses compared to the DiP (24% vs 4%). Compared to cellulose, hemicellulose, as amorphous, low molecular weight polymers, has inferior thermal stability.^72^ The DiP exhibited a strong mass loss rate peak with a maximum at 320 °C, whereas the maximum mass loss rate of BP was at 310 °C.

Thermal properties of RCNFs were found to be only slightly lower compared to the original cellulose pulp. The onset temperature of RCNF1 and RCNF2 was 271 and 280 °C, respectively, being around 10–20 °C lower compared to their corresponding starting materials. Similarly, the maximum mass loss rate for both RCNFs was slightly lower compared to their corresponding raw materials. The onset temperature of CNF was slightly higher compared to RCNF2, sharing the same raw materials. Overall, because of the good thermal stability together with high specific tensile properties, it can be assumed that RCNFs could be used as a reinforcement agent for polymeric composites, which many times request a temperature around 200 °C during the processing.^73^

The cellulose II crystalline structure is described as the thermodynamically most stable cellulose polymorph, and there has been a report that the thermal stability of CNFs^53^ and CNCs^74^ obtained from cellulose II is more stable compared to the corresponding nanomaterials from cellulose I. The higher thermal stability of cellulose II nanomaterials has been ascribed to the stronger hydrogen bonding. However, other factors, such as hemicellulose content, play a significant role in thermal stability as the alkaline mercerization can remove some of the more thermally labile components of cellulose pulp.^53^ Other factors such as DP and the amount of crystalline fractions can have an important role in thermal degradation of cellulose. Previous studies reported that cellulose II from microcrystalline cellulose has a poorer thermal stability compared to the original microcrystalline cellulose.^75^ Here, both RCNFs exhibited slightly lower thermal stability compared to their corresponding raw materials. Both regenerated cellulose samples exhibited slightly lower crystallinity compared to the original pulp (Figure 4b), and the CrI of regenerated cellulose further decreased during the fibrillation due to the strong mechanical force.^76^ The CrI of RCNF1 and RCNF2 was found to be 43% and 42%, respectively. Similarly, the crystallinity of CNFs was notably lower compared to the original pulp (62% of DiP vs 49% of CNFs). Nevertheless, the CrI of CNFs was still somewhat higher compared to RCNFs, which might explain its slightly higher thermal stability. Despite the decreased thermal stability of RCNFs compared to original pulps, their thermal stabilities are higher compared to CNFs produced by using chemical pretreatments.^52,77^ For example, (2,2,6,6-tetramethylpiperidin-1-yl)oxyl-mediated oxidation, postesterification,^78^ and heat-induced conversion of ionic bonds to amide bonds^79^ to produce CNFs have been used to improve thermal stability.

## Conclusions

It was shown that cellulose pulps can be rapidly dissolved in DMSO by adding aqueous TEAOH. Only a minor decrease in DP of cellulose was observed, and the regenerated cellulose was found to be suitable for the production of RCNFs by using mild mechanical disintegration. Thus, the width (around a few nanometers) of the nanofibers produced was in line with more traditional CNFs produced by mechanical disintegration of chemically modified natural cellulose fibers. The RCNFs could be used to produce self-standing films with comparable mechanical properties and higher transparency than that of CNFs produced via intensive mechanical disintegration. Therefore, it was shown that mild, room temperature dissolution and regeneration of cellulose fibers is an efficient pretreatment method to produce a novel type of cellulosic nanomaterial with good properties.

## Supporting Information Available

Photograph of RCNF solutions; TEM images and histograms of the diameters of RCNFs; photograph of reference CNF film (PDF)
