# Supplementary material for: A Fast Dissolution Pretreatment to Produce Strong Regenerated Cellulose Nanofibers via Mechanical Disintegration
Source: Biomacromolecules. 2021 Jul 7;22(8):3366–76. doi: 10.1021/acs.biomac.1c00466 (PMC8382242; doi:10.1021/acs.biomac.1c00466)
Supplement: Supplementary file 1 — bm1c00466_si_001.pdf [file bm1c00466_si_001.pdf]

A fast dissolution pre-treatment to produce strong regenerated cellulose nanofibers by mechanical disintegration

Juho Antti Sirviö,* Matias Lakovaara

Fibre and Particle Engineering Research Unit, University of Oulu, P.O. Box 4300, 90014 Oulu, Finland E-mail: juho.sirvio@oulu.fi.

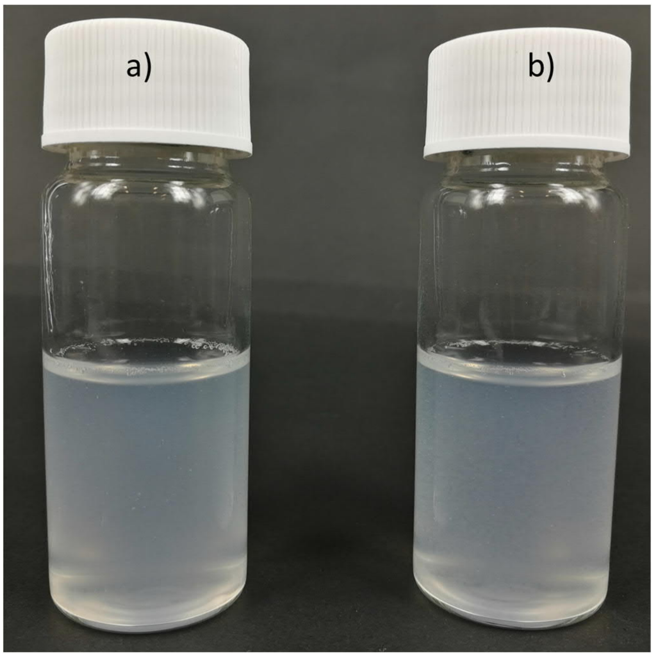

Figure S1. Photograph of a) RCNF1 and b) RCNF2 suspensions

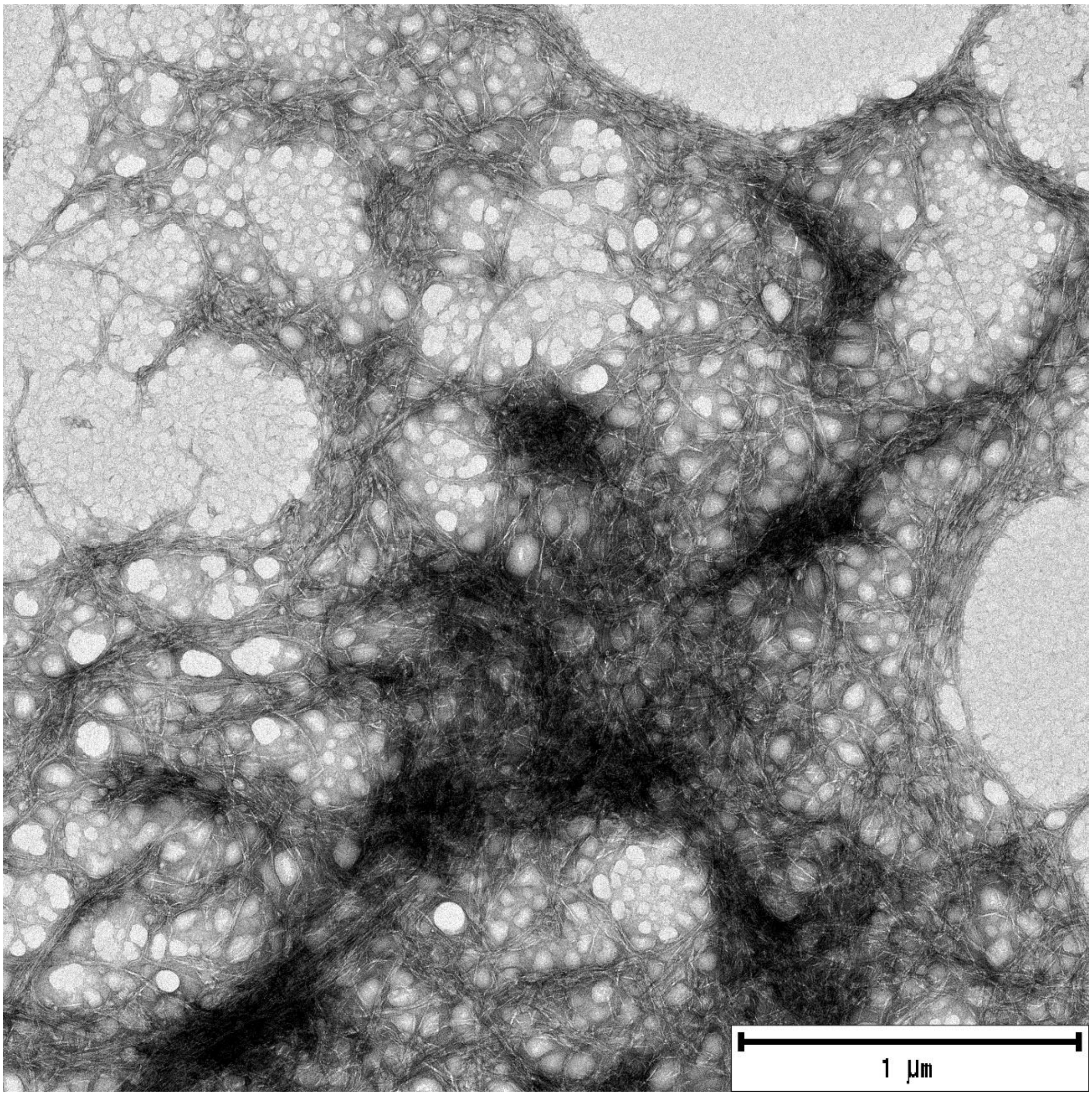
1 µm

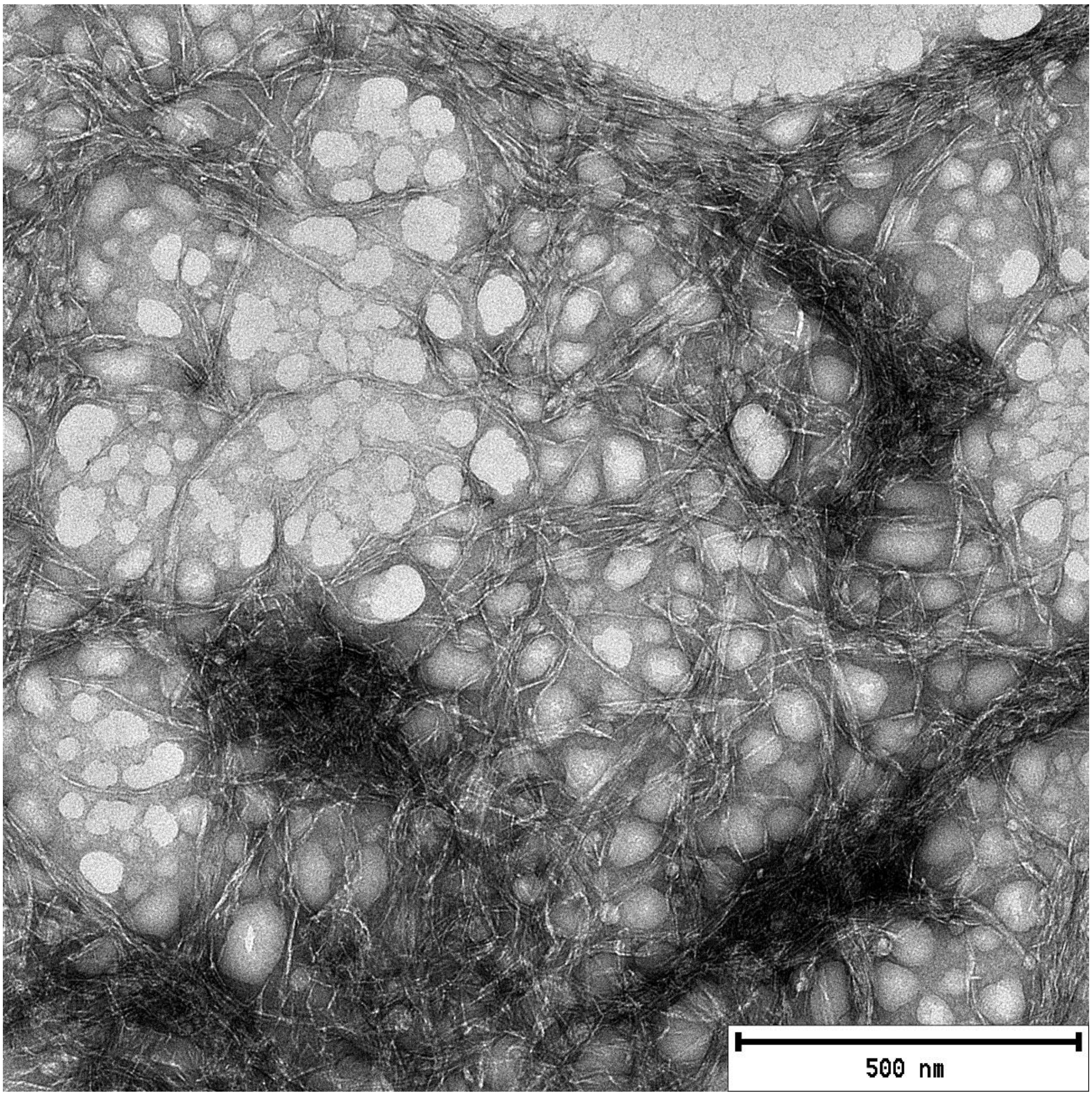
500 nm

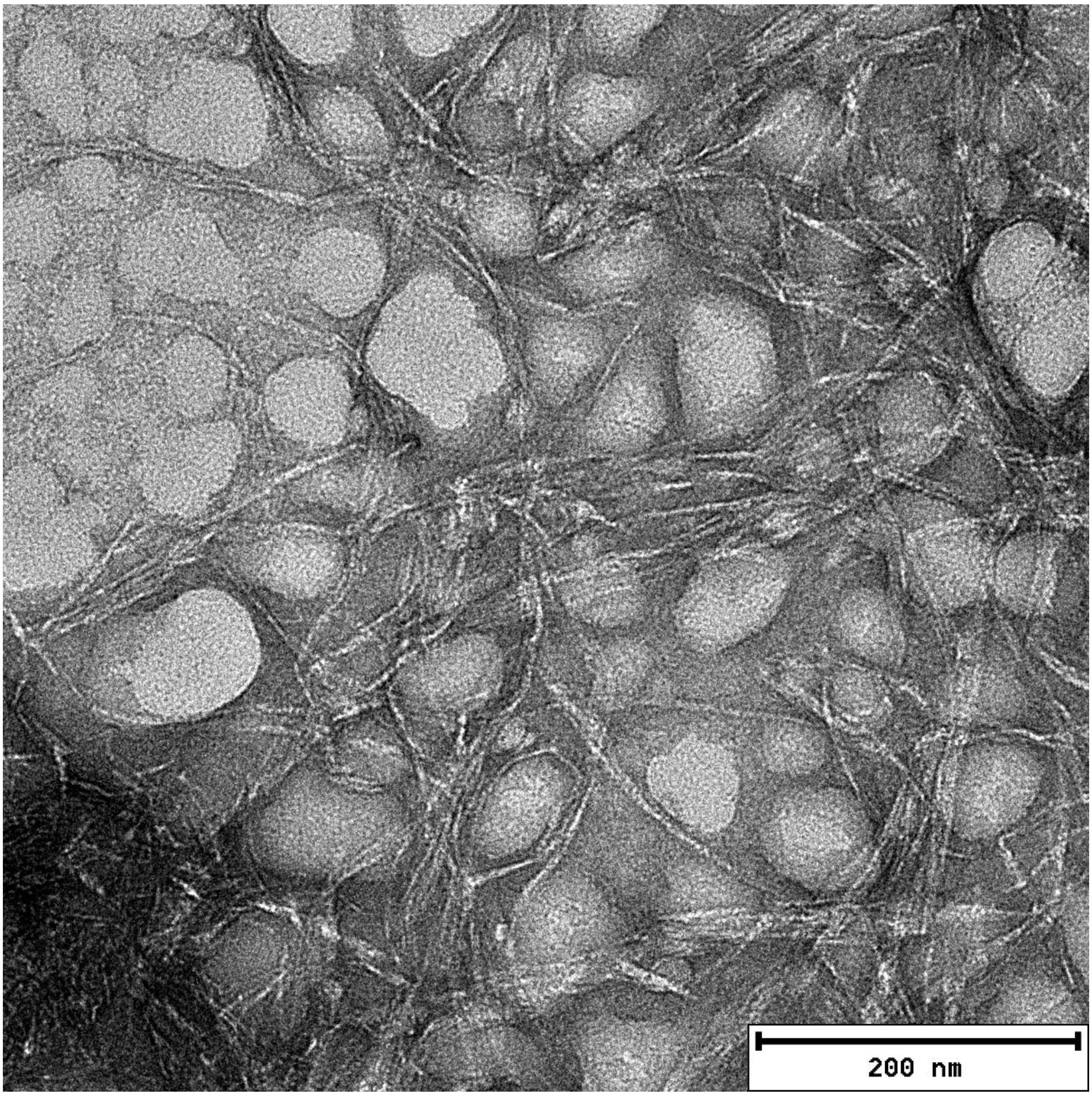
200 nm

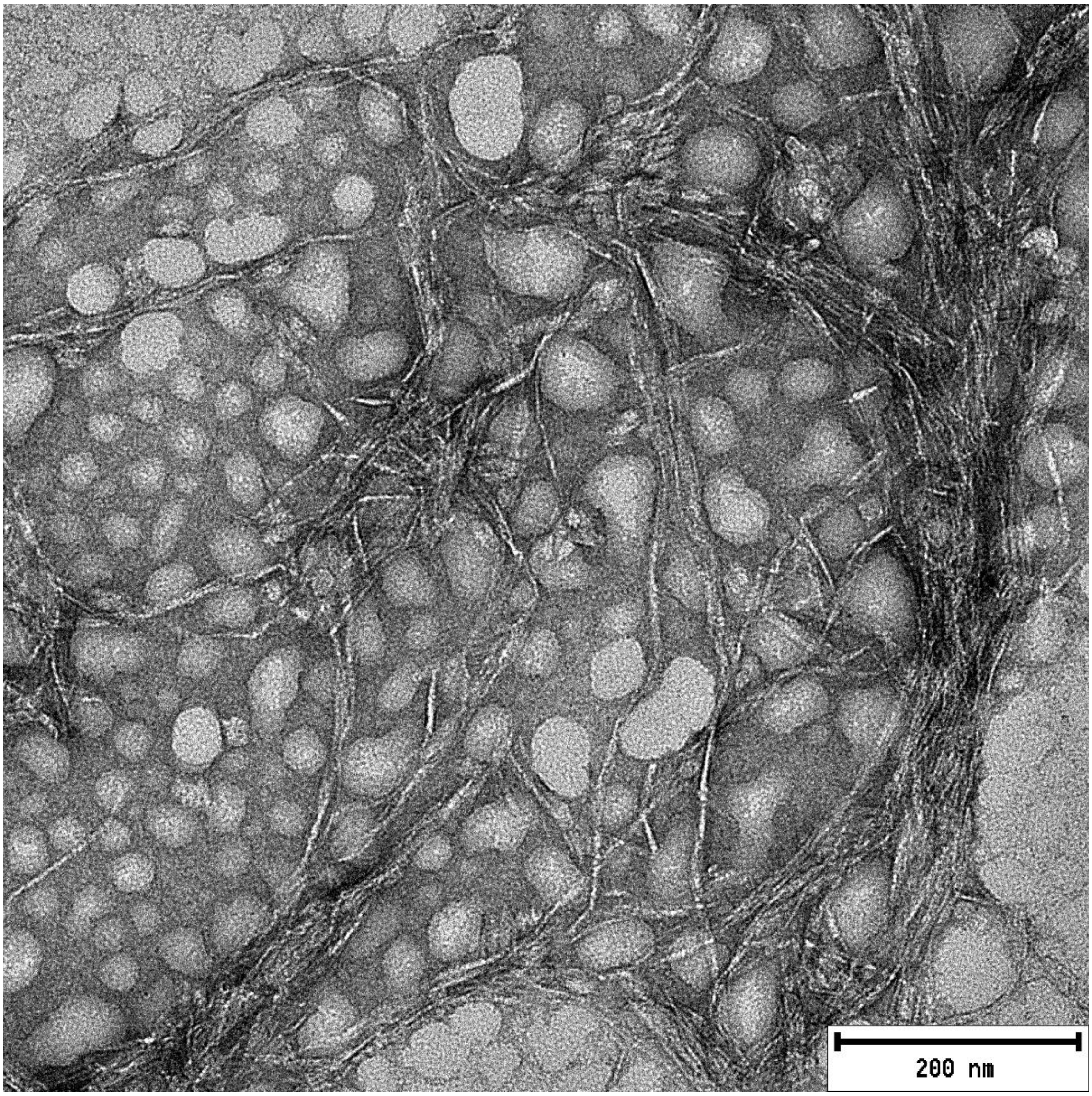

Figure S2. TEM images of RCNF1

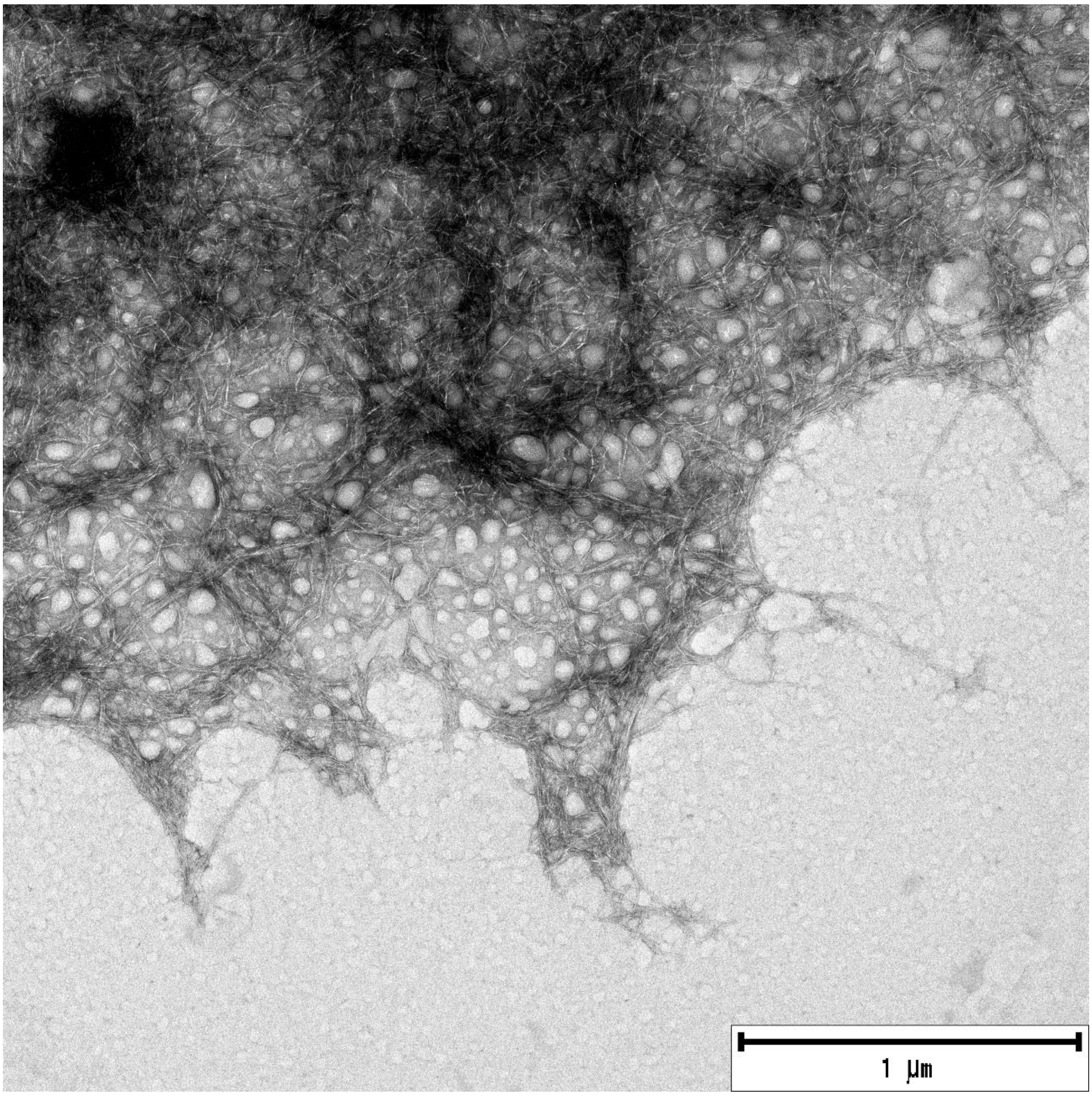
1 µm

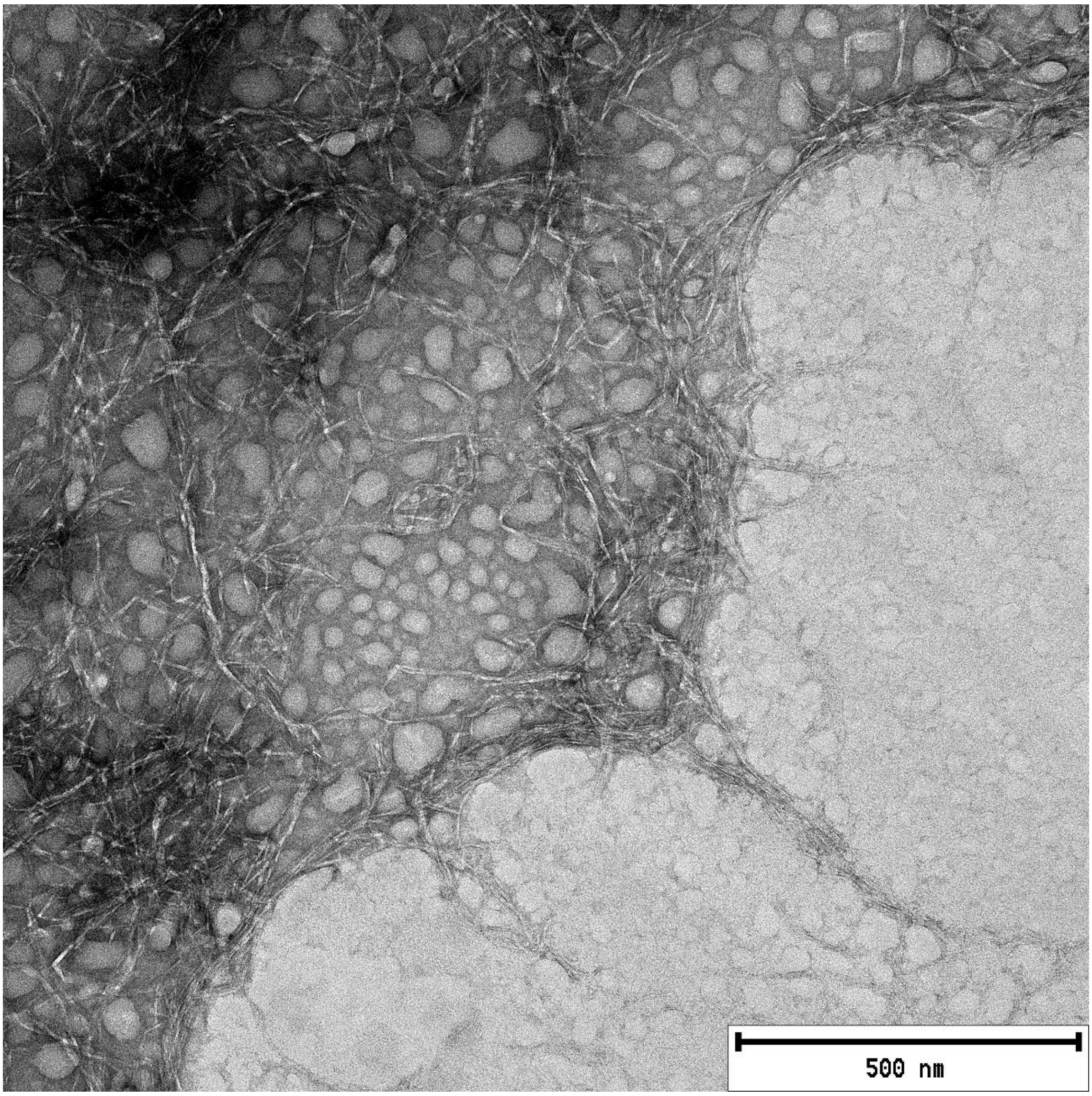
500 nm

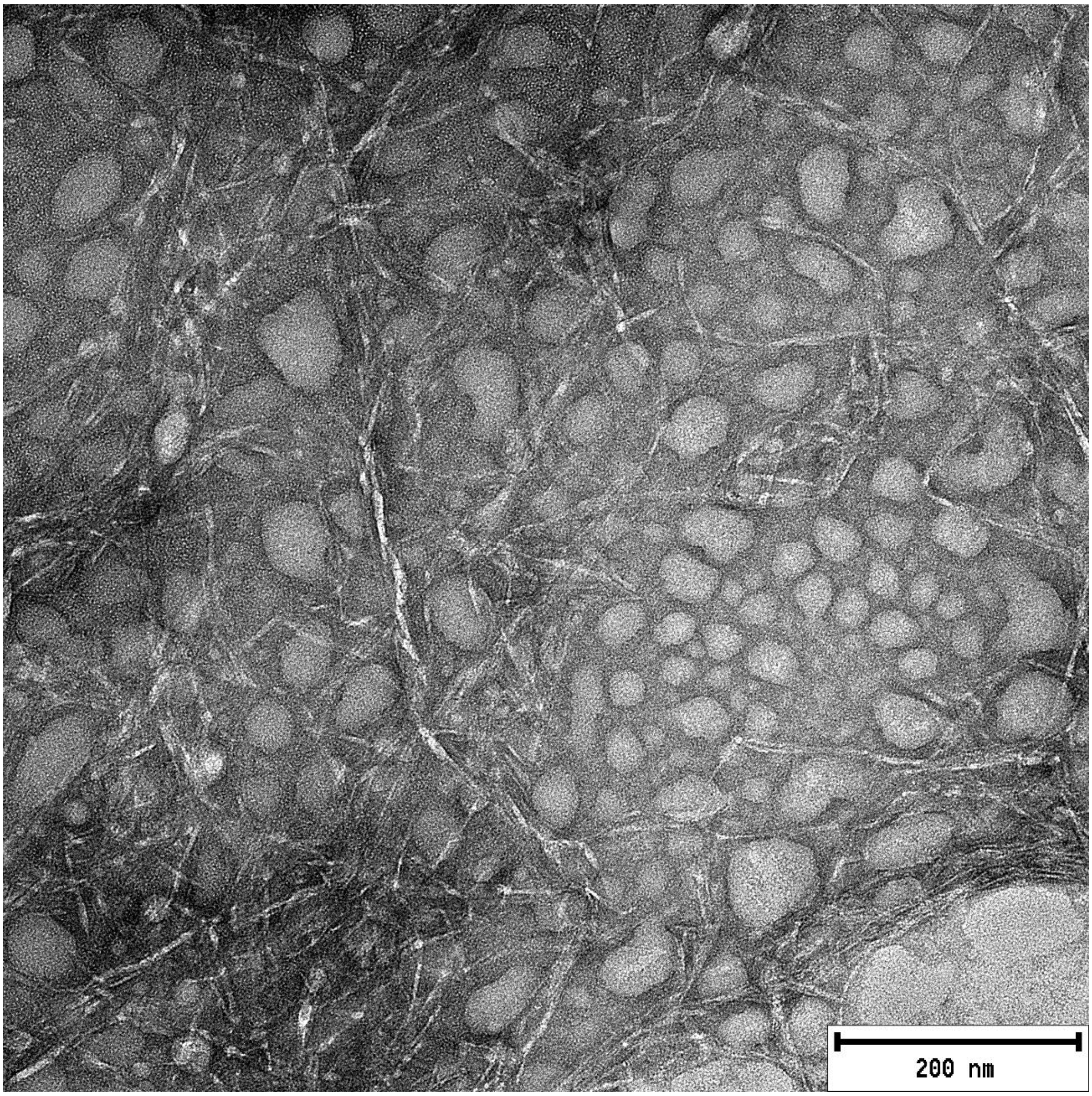
200 nm

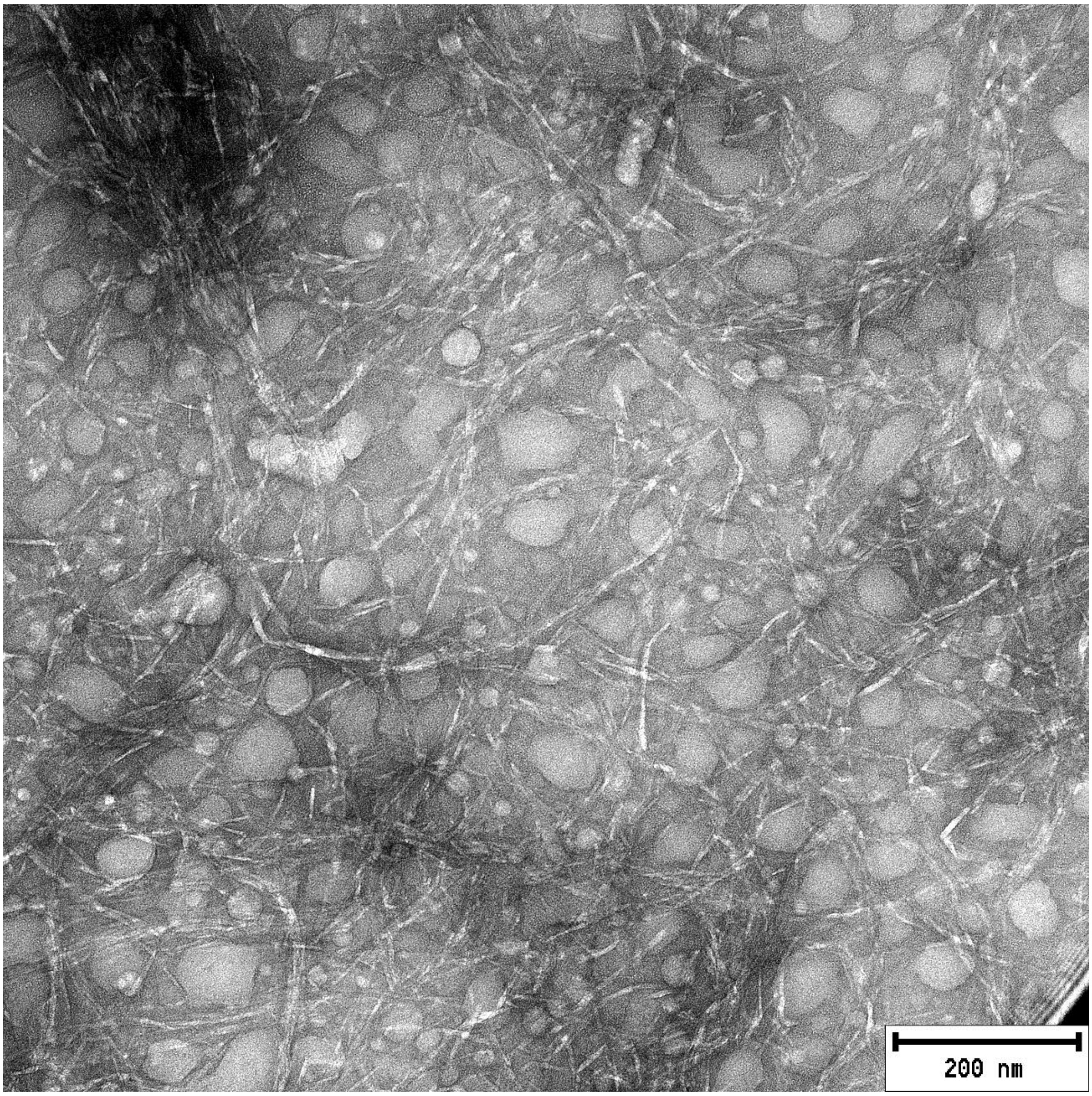

Figure S3. TEM images of RCNF2

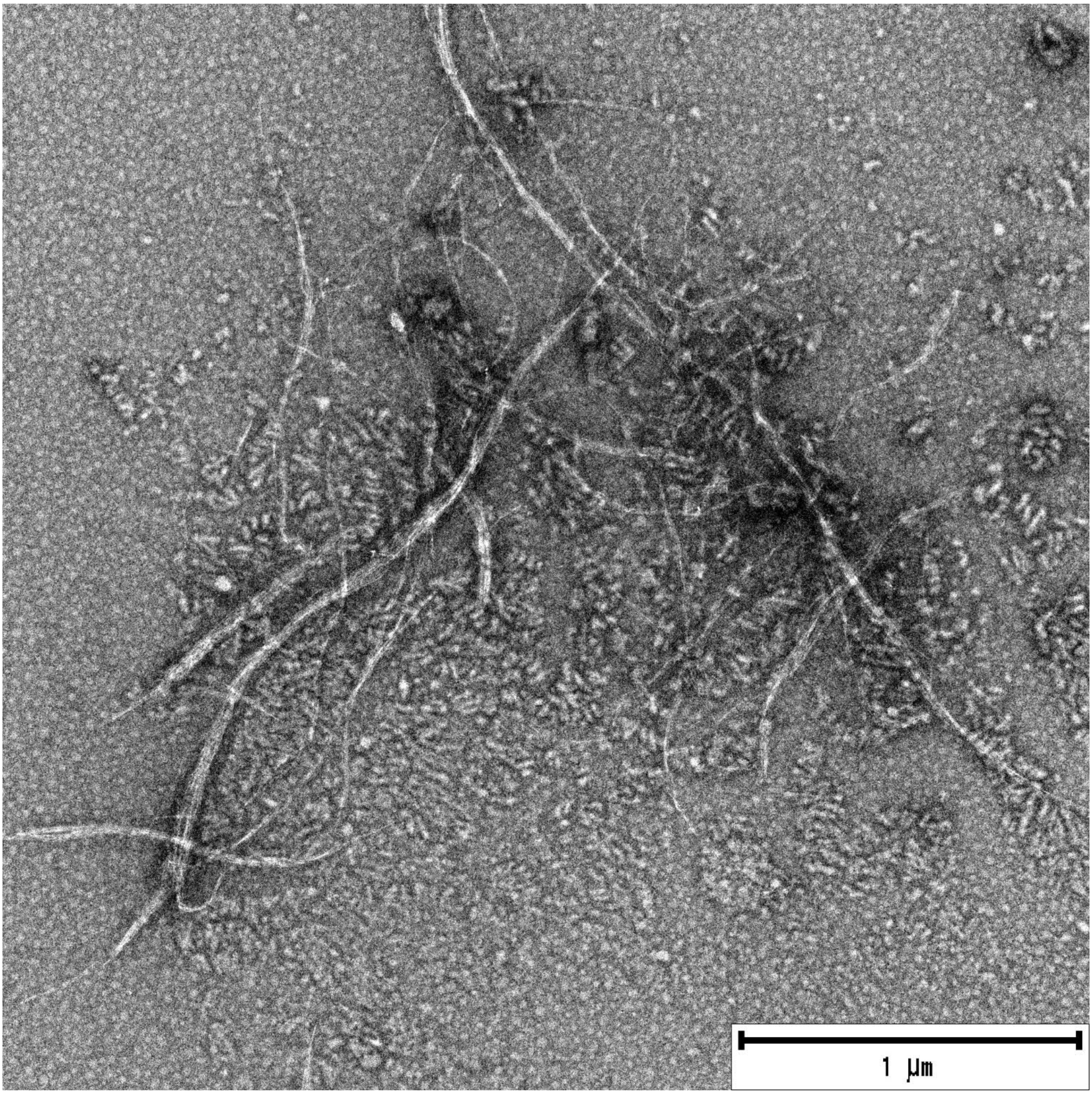
1 µm

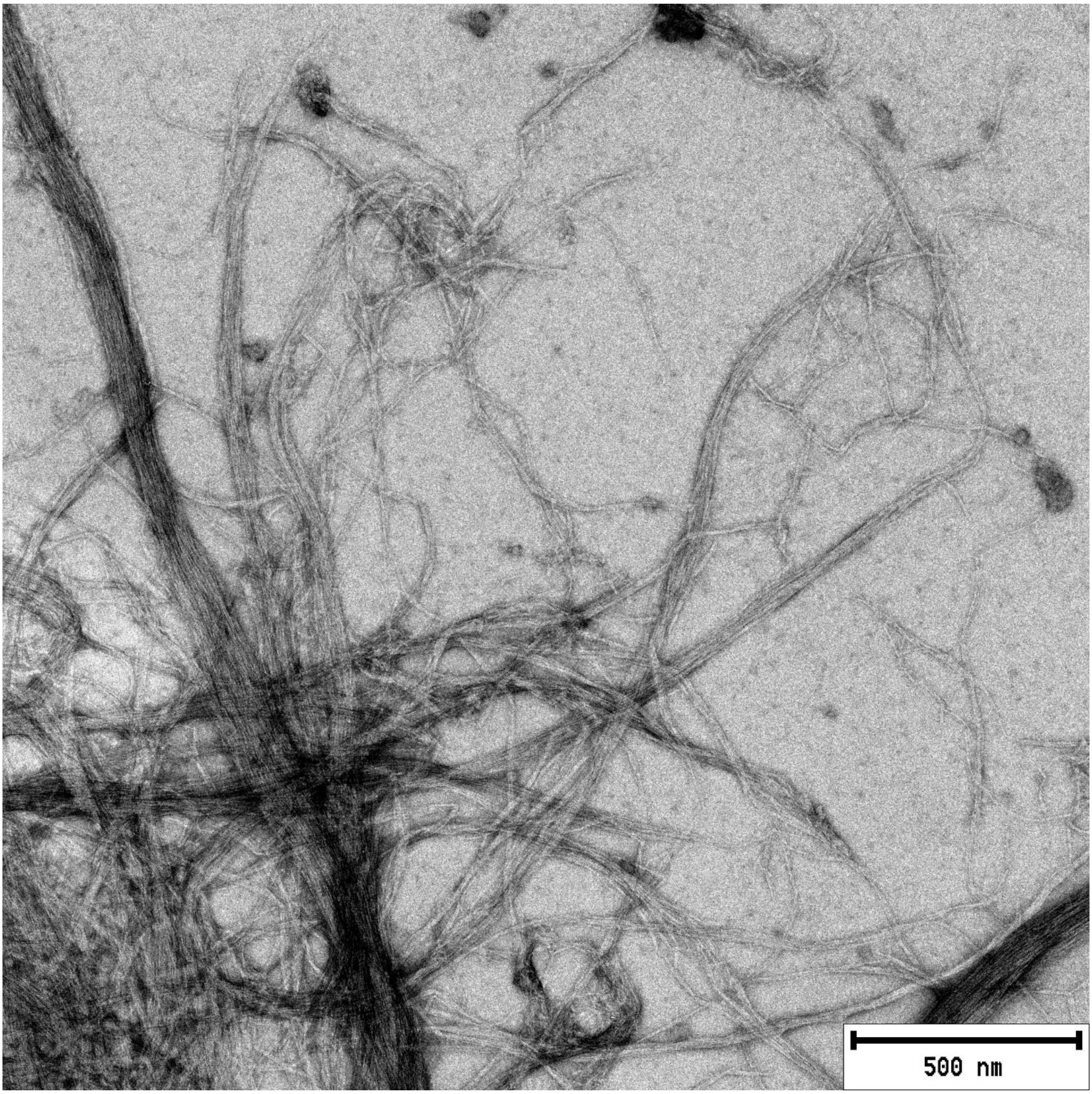
500 nm

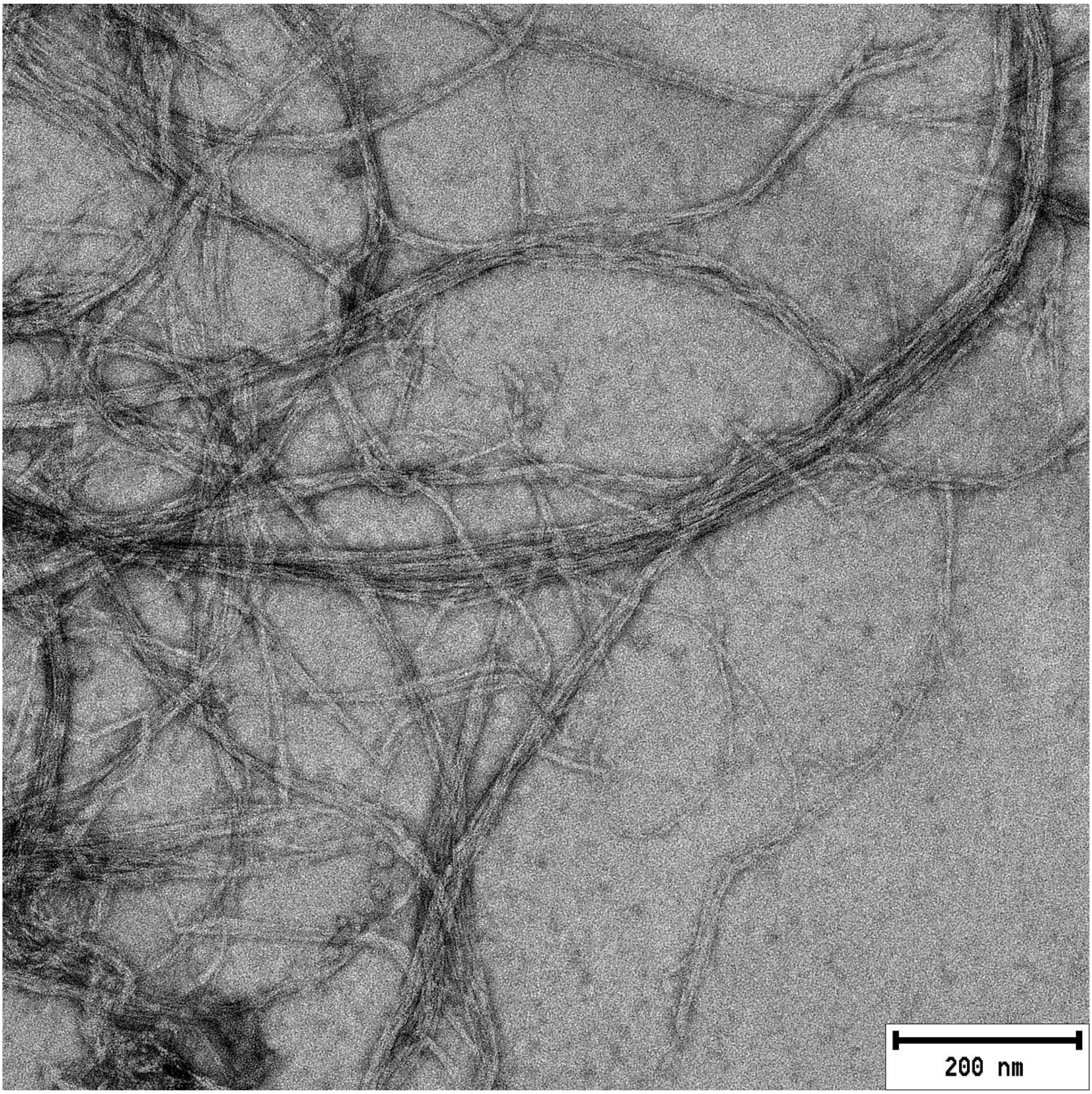
200 nm

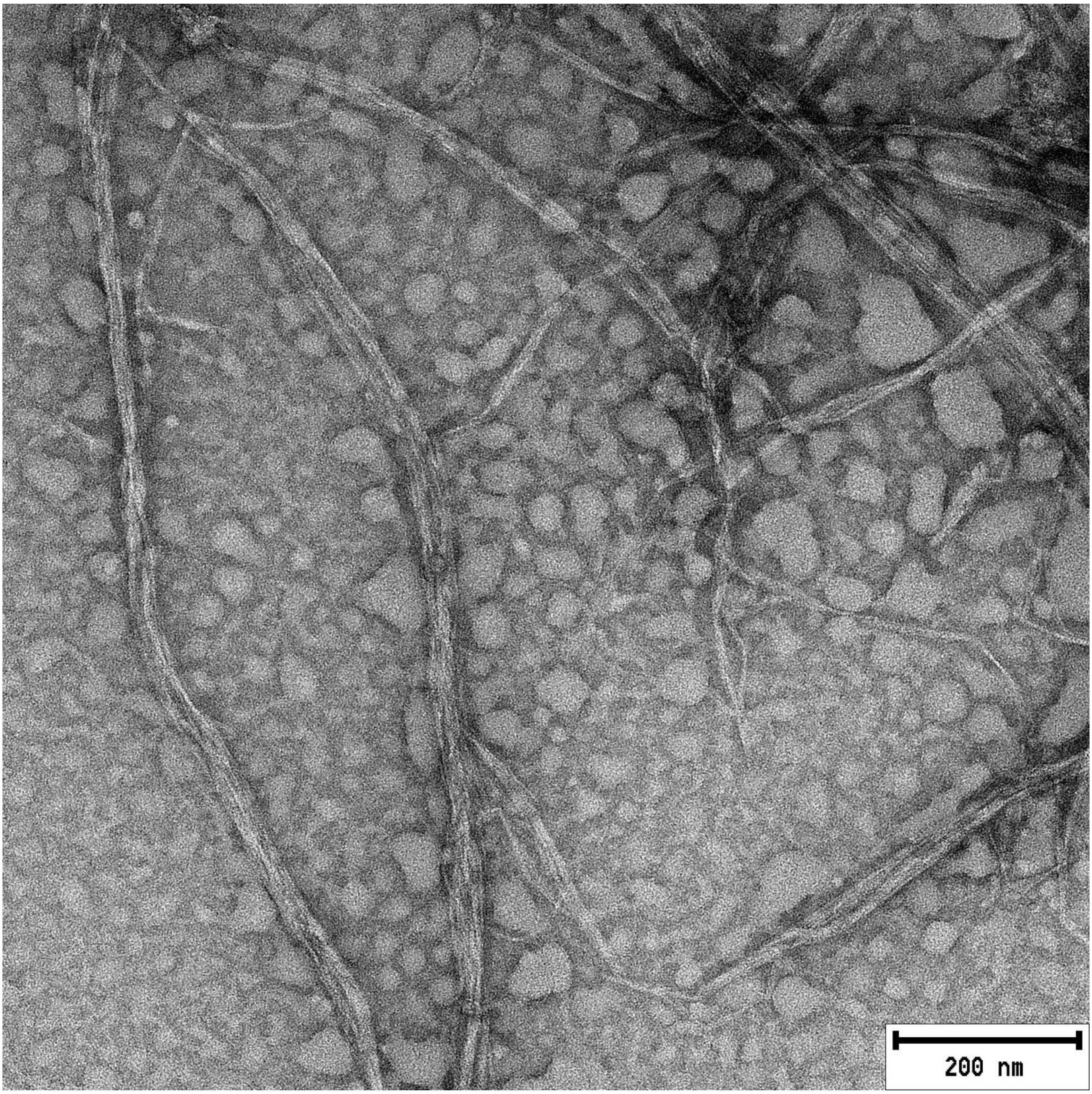

Figure S4. TEM images of reference CNF

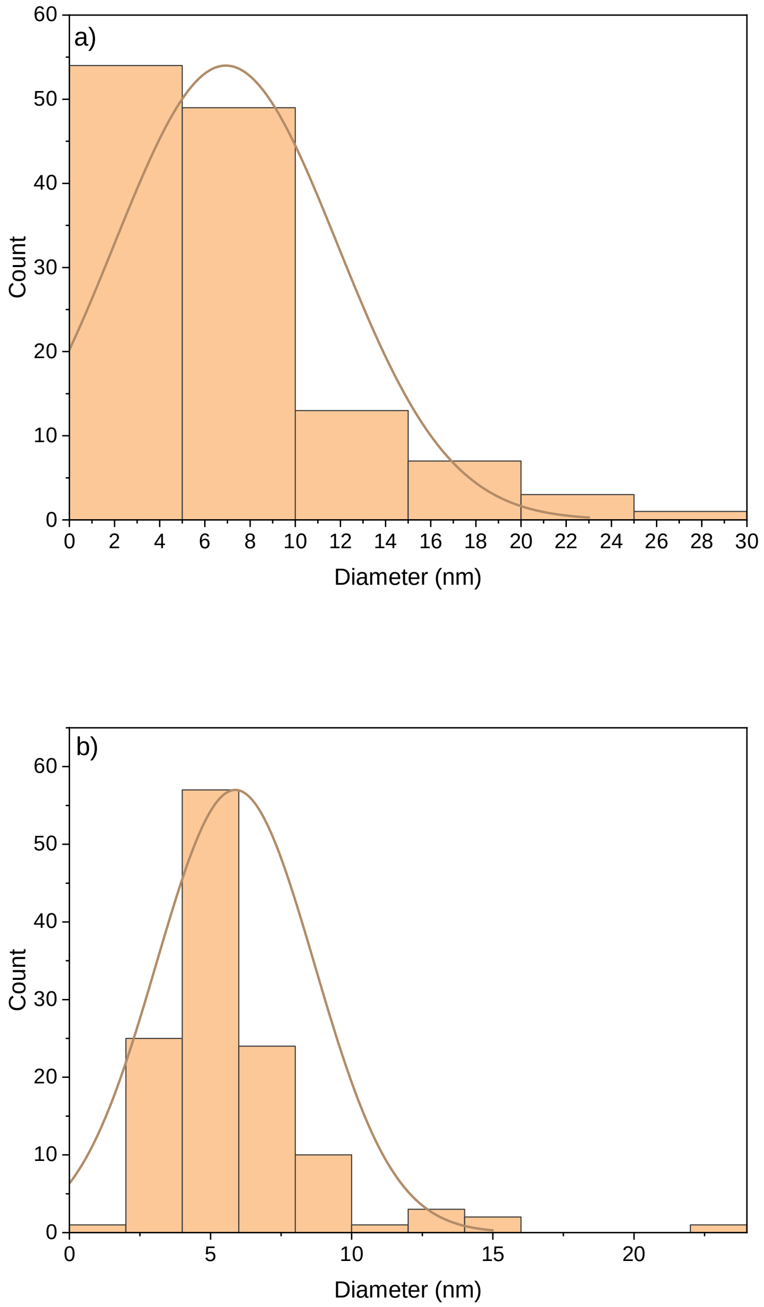

Figure S5. The histograms of the diameters of regenerated cellulose nanofibers: a) RCNF1 and b) RCNF2

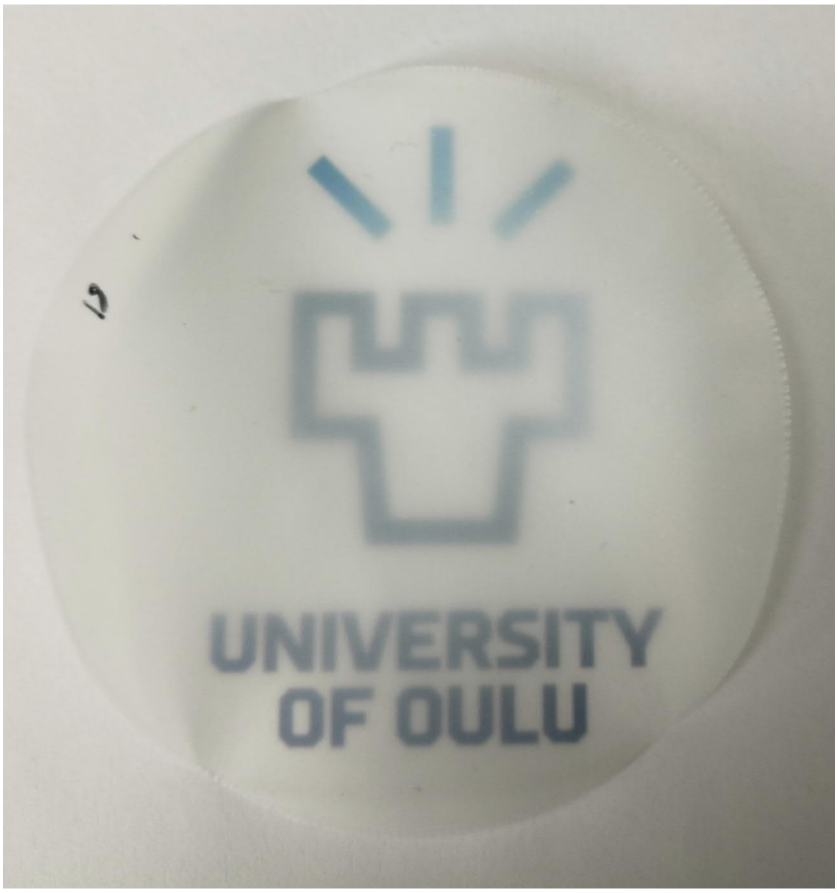

Figure S6. Photograph of the CNF film
